# Multivariate profiling of African green monkey and rhesus macaque T lymphocytes

**DOI:** 10.1038/s41598-019-41209-x

**Published:** 2019-03-18

**Authors:** Wail M. Hassan, Gregory F. Burton, Gabriella A. Pinter, Istvan G. Lauko, Nader N. Mahdi, Mackenzie E. Johnson

**Affiliations:** 1 Department of Biomedical Sciences, University of Missouri Kansas City School of Medicine, Kansas City, MO USA; 2 Department of Biomedical Sciences, University of Wisconsin – Milwaukee, Milwaukee, WI USA; 3 Department of Chemistry and Biochemistry, Brigham Young University, Provo, UT USA; 4 Department of Mathematical Sciences, University of Wisconsin – Milwaukee, Milwaukee, WI USA

## Abstract

The complexity of immune responses limits the usefulness of univariate methods in answering complex immunology questions. To demonstrate the utility of a multivariate approach, we employ such approach to compare T cells of African green monkeys (AGMs) and rhesus macaques (RMs). Among the most prominent distinguishing features we found were lower CD3 and higher CD28 surface expression in AGMs compared to RMs. After *in vitro* stimulation, a larger proportion of AGM T cells secreted cytokines, especially those producing more than one cytokine (i.e. multifunctional cells). To find out whether multifunctional responses associate with protection in other species, we compared T cells of cynomolgus macaques (CMs) infected with wild-type Simian Immunodeficiency Virus (SIV) to those of CMs infected (vaccinated) with a replication-defective virus. Wild-type SIV infection in macaques leads to simian Acquired Immunodeficiency Syndrome (AIDS), which does not happen in animals previously vaccinated with a replication-defective virus. Interestingly, after *in vitro* stimulation, multifunctional cells were more abundant among T cells of vaccinated CMs. Our results propose T-cell multifunctionality as a potentially useful marker of immunity, although additional verification is needed. Finally, we hope our multivariate model and its associated validation methods will inform future studies in the field of immunology.

## Introduction

The current study aims to demonstrate the utility of multivariate data analysis in studying complex immunological variables. To date, the majority of studies employ a univariate approach to the study of immunology. No doubt, univariate studies have shown admirable success in building our knowledge of the immune system as we know it today. Using this knowledge, it was possible to define simple patterns of protective immunity, such as immunity against hepatitis B virus^1^ and exotoxins of *Clostridium tetani* and *Corynebacterium diphtheriae*^2^. In these infections, an antibody, in high enough concentrations, seems to be sufficient to protect against disease. However, in the context of infections where more complex immune responses may be required for protection, such as lentiviral infections, a multivariate approach is needed to enable the recognition and exploration of complex patterns, rather than focusing on individual variables. A detailed commentary on the use of multivariate data analysis in immunological studies has been published by Bernd Genser and coauthors^3^.

Central to our multivariate approach is the use of principal component analysis (PCA). The rationale of using PCA was to enable the simultaneous evaluation of many variables. In studies dictating the simultaneous evaluation of more than three variables, as is the case in the current study, graphical presentation of data points is impractical without computational data reduction. Data reduction can be accomplished by condensing measured variables into a smaller number of computed variables. PCA is a statistical method that does just that by merging correlated variables into linearly uncorrelated “artificial variables”. Graphical display of subjects based on the new, condensed variables — more formally known as principal components (PCs) — enables visual inspection of the data. It is important to realize that PCs are not calculated with pre-existing, user-defined groups in mind. Therefore, PCA enables unbiased exploration of the “natural” partitioning of data points. Furthermore, the relative contributions of each of the variables to the discrimination between groups of subjects are determined, highlighting the most useful variables in discriminating between groups. Our multivariate approach also includes statistical testing to verify the appropriateness of using PCA, the adequacy of sample size and the significance of PCs. Another similar method used in this study is multidimensional scaling (MDS). MDS computation is identical to that of PCA, except that the input into PCA is a covariance or a correlation matrix, while MDS uses a similarity matrix describing the interrelatedness between study subjects as an input. In addition, we use hierarchical clustering to show the relatedness of study subjects in the form of a tree (also known as a dendrogram). By using different, in a sense independent, statistical methods to test the partitioning of study subjects, we hope to demonstrate congruence of conclusions across analytical methods, thus, increasing confidence in our data interpretation.

In this study, we compared T cells isolated from healthy AGMs to those isolated from healthy RMs in the absence of any known infections. Interest in the two animal species stems from their extensive use as model organisms, especially in the area of Human Immunodeficiency Virus (HIV) and AIDS^4,5^. More importantly, AGMs are one of the natural hosts of SIV, which do not develop AIDS as a result of the infection^6,7^, while RMs develop an AIDS-like syndrome upon experimental infection^7^. The goal of this study is twofold. First, we wanted to demonstrate the utility of multivariate analysis in interrogating immunological questions. Secondly, we wanted to identify the variables with the greatest power to distinguish between AGMs and RMs among a set of phenotypic and functional variables mainly focusing on CD4^+^ T cells. We compared the abundance of T-cell subsets between the two species; both cell count per cubic millilitre of whole blood and the percentage of each cell population that falls within each of its constituent subdivisions were measured. We also compared expression of important T-cell surface proteins and post-stimulation cytokine-secretion patterns. Additionally, we developed an immune-profiling scheme composed of multivariate analyses (e.g. PCA and MDS) and additional confirmatory tests [e.g. Bartlett’s test of sphericity, Kaiser-Meyer-Olkin measure of sampling adequacy (KMO) and Monte Carlo simulation] that we hope will inform future studies. Likely applications of multivariate immune profiling may include efficacy evaluation of experimental vaccine candidates, testing batch-to-batch variations of licensed vaccines and evaluation of the immune status of an individual against specific infectious agents.

Finally, we wanted to explore the potential significance of the unique features we identified in AGMs, which separated them from RMs. A couple of questions emerged: (1) are these differences merely species-specific variations that has no generalizable applications in other species? (2) do these differences bear any resemblance to the features that separate pathogenic from non-pathogenic SIV infections in susceptible host species? To address these questions, we present a comparison between wild-type (pathogenic) and replication-deficient (non-pathogenic) SIV infection in cynomolgus macaques (CMs).

## Results

### Profiling peripheral blood T-cell subpopulations

The current study is mainly focused on CD4^+^ T cells, but before exclusively focusing on these cells, we wanted to examine their abundance relative to the rest of T-cell subsets in both AGMs and RMs. The first set of variables examined was related to the distribution and abundance of various T-cell populations. We used blood samples from 8 AGMs and 19 RMs throughout the study, unless otherwise indicated. We classified T cells — defined as CD3^+^ lymphocytes — based on the expression of specific surface proteins. T cells were divided into CD4^+^, CD8^+^, double positive (CD4^+^ CD8^+^) and double negative (CD4^−^ CD8^−^) based on surface expression of the glycoproteins CD4 and CD8. Each of these T-cell subsets was further subdivided into three compartments: central memory (CD28^hi^CD95^+^), effector memory (CD28^−^CD95^+^) and naïve (CD28^lo^CD95^−^) based on surface expression of CD28 and CD95 (Supplemental Fig. S1).

**Figure 1:**
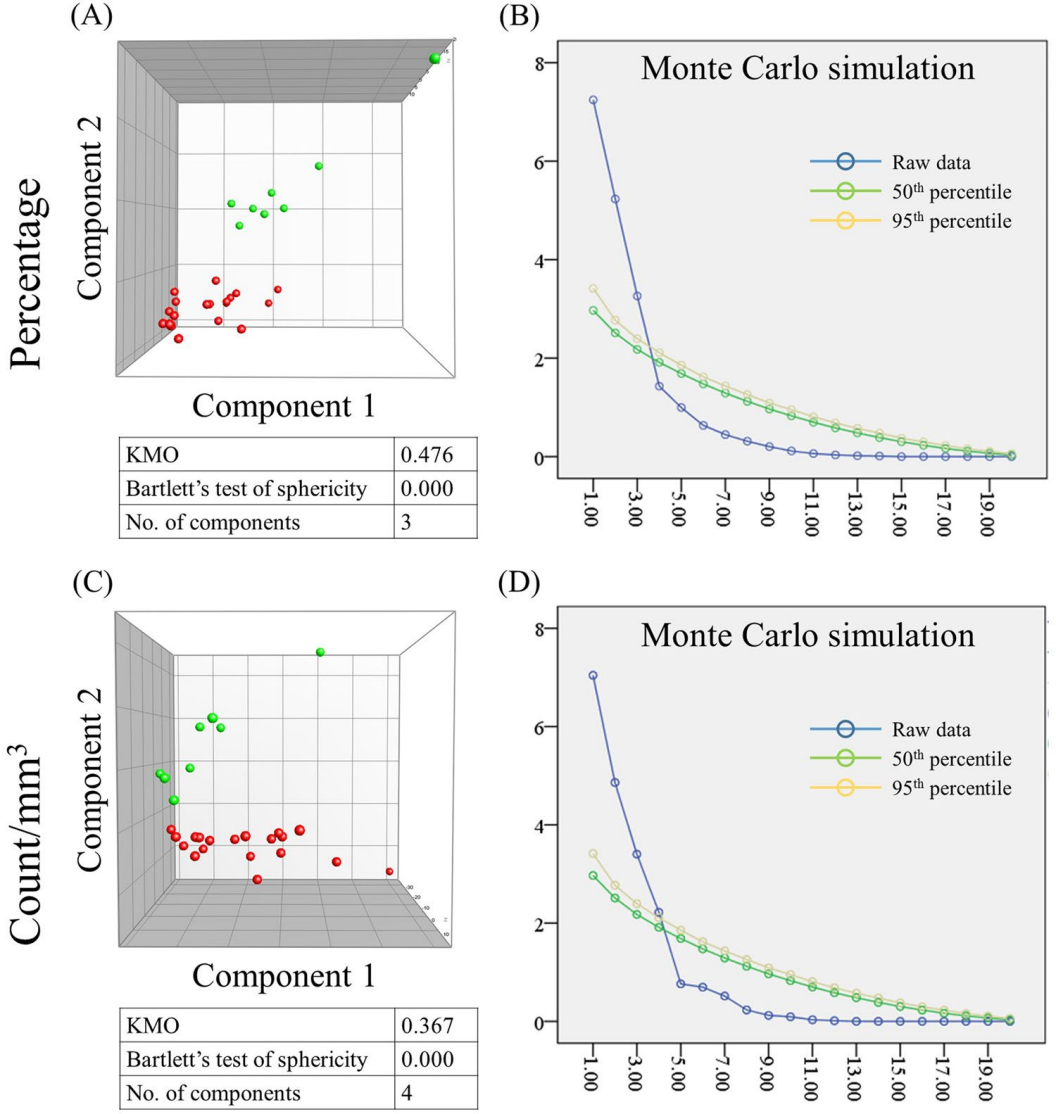
Segregation of African green monkeys (green; n = 8) and rhesus macaques (red; n = 19) based on peripheral blood T-cell subsets. Total T cells, CD4^+^, CD8^+^, CD4^+^ CD8^+^ (double positive) and CD4^−^ CD8^−^ (double negative) T cells were expressed as percentage of the corresponding parental cell population and as absolute counts per cubic millilitre of whole blood. The animals were plotted using the coordinates of the top two principal components using percent (**A**) and absolute count (**C**) data. Scree plots show the eigenvalues (y axis) corresponding to each principal component (x axis). Raw eigenvalues as calculated in principal component analysis (blue) and eigenvalues modelled by Monte Carlo simulation at 50^th^ and 95^th^ percentile confidence (green and yellow, respectively) are shown for percent (**B**) and absolute count (**D**) data. Principal components were considered significant if their raw eigenvalues exceeded the corresponding modelled 95^th^ percentile values. Kaiser-Meyer-Olkin measure of sampling adequacy (KMO), Bartlett’s test of sphericity *p* values and the number of significant components are indicated below each plot (an explanation of the statistical tests is discussed in the methods section).

We described each of these cell populations by two types of variables: the population’s cell count per cubic millilitre of whole peripheral blood (henceforth referred to as “absolute count”) and percentage it makes up in its parental cell population (henceforth referred to as “percent”). For example, by percent naïve T cells and percent naïve CD4^+^ T cells, we refer to the percentage of these specific cell populations in their respective parental populations; in this case all T cells and all CD4^+^ T cells, respectively. We applied PCA to explore untrained, natural groupings of animals based on the aforementioned variables. In doing so, the animals were plotted solely based on the data variables, with no influence from antecedent knowledge of the species or other user-defined groups. Knowing the species of each subject, however, we were able to observe whether African green and rhesus monkeys were segregated in the resulting plots. We found that, indeed, the two animal species were almost completely segregated using both absolute count and percent variables, although a slightly better segregation was observed using the latter (Fig. 1).

As explained in more detail in the methods section, Bartlett’s test of sphericity, KMO and Monte Carlo simulation were used to validate our PCA. For the T-cell populations’ dataset, KMO was low, indicating a small size and prompting us to look at the data in different ways in an attempt to validate our results. We used two additional methods: MDS and hierarchical clustering. Like PCA, both methods explore natural grouping of the data, with no regard to user-defined groups. Using both methods, AGMs and RMs were completely separated (Supplementary Fig. S2), which is consistent with the results obtained by PCA.

Next, we were curious to know which of the T-cell subsets contributed the most towards separating the two species, AGMs and RMs. For this purpose, we examined the contribution of each of the variables to the principal component responsible for the segregation of the two species — principal component 2 for both percent and absolute count data (Fig. 1). We found that the most discriminatory absolute count variables were, in descending order, effector memory CD8^+^, total double negative, effector memory double positive, naïve double positive and effector memory CD4^+^ T cells, while the most discriminatory among percent variables were central memory double negative, effector memory CD8^+^, naïve double positive, naïve double negative and central memory double positive T cells (Table 1). It is worth noting that AGMs and RMs were not segregated on the coordinate of the first principal component — the principal component accounting for the majority of variability in the dataset. Instead, using both percent and absolute count data, the two species were segregated on the coordinate of the second principal component (Fig. 1), implying that, although discriminatory variations were sufficient to separate the two species, most of the variation in T-cell subpopulations were actually not discriminatory.

**Table 1:** Variable contributions to the principal components responsible for the greatest segregation between African green monkeys (n = 8) and rhesus macaques (n = 19).

Percent data		Count data	
Variable	PC2 (9.5%)	Variable	PC2 (10.4%)
**Central memory double negative T cells**	**2.96**	**Effector memory CD8** ^**+**^ **T cells**	**3.60**
**Effector memory CD8** ^**+**^ **T cells**	**2.57**	**Total double negative T cells**	**3.39**
**Central memory double positive T cells**	**2.11**	**Effector memory double positive T cells**	**2.93**
Effector memory double negative T cells	1.83	Effector memory double negative T cells	1.42
Effector memory T cells	1.41	Effector memory T cells	1.29
Effector memory double positive T cells	1.36	Central memory double negative T cells	0.49
Total double negative T cells	0.61	Central memory double positive T cells	0.44
Total T cells	0.56	Central memory CD8^+^ T cells	0.37
Central memory CD8^+^ T cells	0.49	Total T cells	0.34
Total CD8^+^ T cells	0.09	Total double positive T cells	0.06
Central memory T cells	0.08	Total CD4^+^ T cells	−0.34
Naïve CD4^+^ T cells	−0.04	Central memory T cells	−0.38
Effector memory CD4^+^ T cells	−0.68	Naïve CD8^+^ T cells	−0.62
Naïve T cells	−0.88	Total CD8^+^ T cells	−0.64
Naïve CD8^+^ T cells	−1.21	Naïve T cells	−1.02
Total CD4^+^ T cells	−1.31	Naïve double negative T cells	−1.20
Central memory CD4^+^ T cells	−1.83	Central memory CD4^+^ T cells	−1.56
Total double positive T cells	−1.87	Naïve CD4^+^ T cells	−1.60
**Naïve double negative T cells**	**−2.16**	**Effector memory CD4** ^**+**^ **T cells**	**−1.83**
**Naïve double positive T cells**	**−2.25**	**Naïve double positive T cells**	**−2.08**

Using percent or absolute count data, the principal component responsible for the most segregation between the two animals was the second (PC2). Variance explained by PC2 is shown between parentheses. The most contributing variables in each column are shown in bold.

### Profiling surface markers’ expression of peripheral blood T-cell subsets

**Figure 2:**
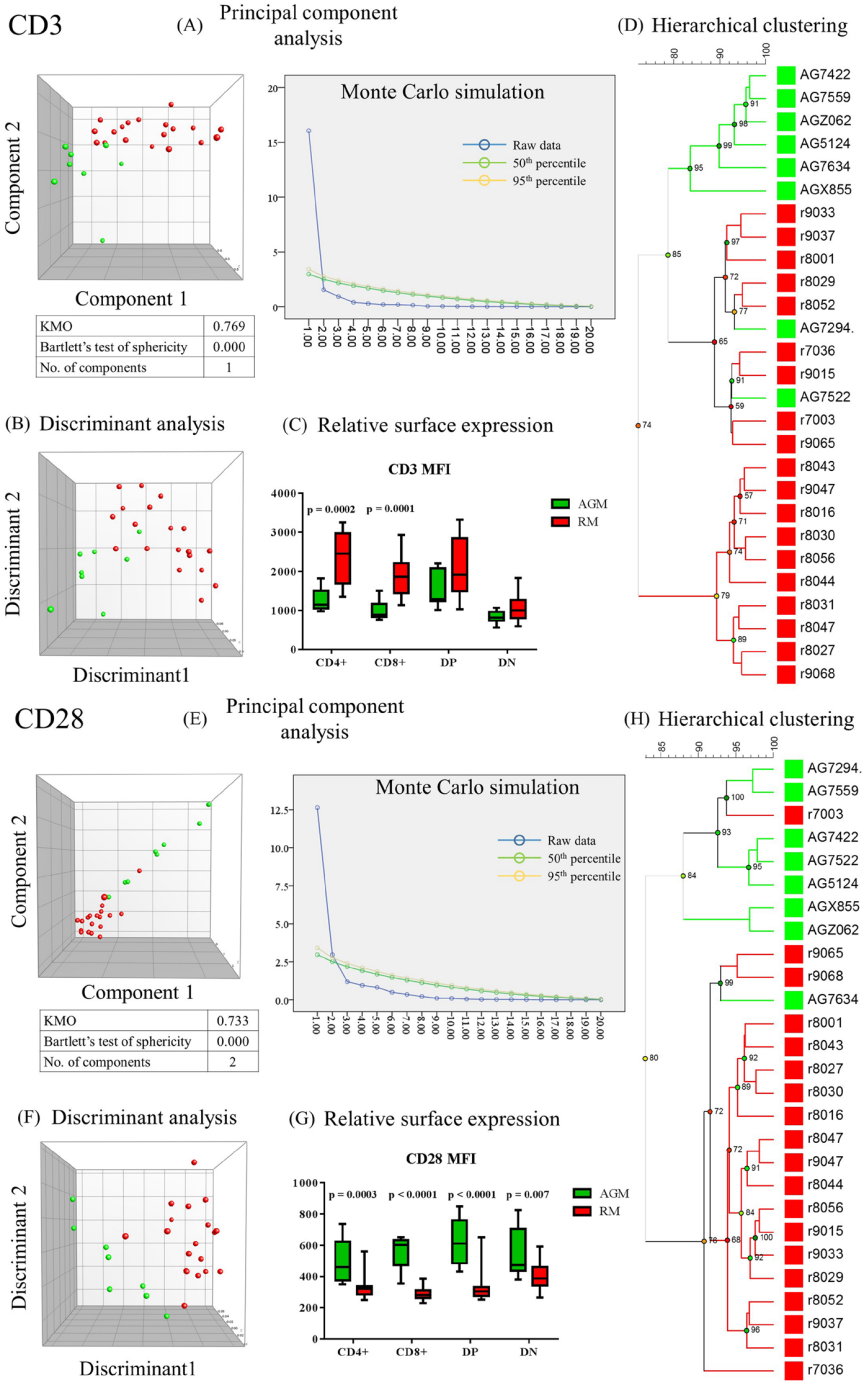
Segregation of African green monkeys (AGMs; green; n = 8) and rhesus macaques (RMs; red; n = 19) based on mean surface expression of CD3 and CD28 by peripheral blood T lymphocytes. After carrying out principal component analysis and multidimensional scaling, individual animals were plotted using principal components 1 and 2 (**A**,**E**) or discriminants 1 and 2 (**B**,**F**). Scree plots show raw eigenvalues and eigenvalues modelled at 50^th^ and 95^th^ percentile (blue, green and yellow, respectively) for each principal component (**A**,**E**). Components with higher raw eigenvalues than the corresponding 95^th^ percentile modelled values were considered significant components. Kaiser-Meyer-Olkin measure of sampling adequacy (KMO), Bartlett’s test of sphericity p values and the number of significant components are indicated below each plot. Relative CD3 and CD28 expression levels on CD4^+^, CD8^+^, double positive and double negative T cells are shown (**C**,**G**). Statistical significance of differences between African green monkeys and rhesus macaques CD3 and CD28 expression was determined using multiple t-tests with Holm-Sidak correction for multiple comparisons. P values below 0.05 are indicated. Hierarchical clustering was performed on CD3 (**D**) and CD28 (**H**) profiles. Multidimensional scaling and hierarchical clustering were based on similarities between individual animals as calculated using Canberra distances. Representative dot plots are shown in Fig. S3.

Surface proteins play essential roles in T cell function. Quantitative differences in cell surface expression of these molecules influence T cell survival and function. We quantified surface expression of CD3, CD4, CD8, CD25, CD28 and CD95 by measuring the average brightness of cells after staining them with specific fluorescently-labelled monoclonal antibodies. Representative dot plots depicting relevant flow cytometry data and fundamental gating strategies are shown in supplemental Fig. S3. Surface expression of each of these proteins was evaluated on all T-cell subsets that express them, resulting in a profile for each surface molecule. As we tested the effectiveness of each individual surface molecule’s profile in distinguishing between African green and rhesus monkeys, we identified CD3 and CD28 as best distinguishing features between the two animal species. Neither of the two molecules, however, resulted in complete segregation between the species (Fig. 2). The use of the CD8 profile resulted in less effective, yet visually discernible, separation. CD4, CD25, CD28 and CD95 profiles failed to effectively distinguish between AGMs and RMs (Supplemental Fig. S4). Interestingly, using the combined profiles of all molecules resulted in complete separation of the two animal species using PCA, MDS and hierarchical clustering. However, the number of variables — ninety one — was too large for the sample size tested and neither KMO nor Bartlett’s sphericity test *p* value was possible to calculate (Supplemental Fig. S4). Even more interesting is that combining the best discriminators (i.e. CD3 and CD28 or CD3, CD8 and CD28) did not lead to the complete separation observed when all six profiles were combined (data not shown). We ranked all variables by their contribution to principal component 2 to define the most discriminatory variables. Not surprisingly, the top most discriminatory variables were from CD3 and CD28 profiles. CD28 surface expression of total double positive, total CD8^+^ T cells and total T cells ranked 1^st^, 5^th^ and 10^th^, respectively. CD3 surface expression of total CD8^+^ T cells, central memory T cells and central memory CD4^+^ T cells ranked 2^nd^ through 4^th^. The top 23 variables were all related to CD3 or CD28 expression (Table 2). After identifying the best discriminatory of the phenotypic variables described above, we became interested in exploring functional T cell characteristics to identify the most discriminatory among them. For this reason, we studied cytokine-secretion patterns in AGM and RM’s T cells upon *in vitro* stimulation.

**Table 2:** Variable contributions to the most discriminatory principal component in a principal component analysis investigating surface expression of T cell surface proteins in African green monkeys (n = 8) and rhesus macaques (n = 19).

Rank	Surface protein	CD4/CD8 subset	Memory/naïve compartment	PC2 (3.15%)
1	CD28	Double positive	Total	1.73
2	CD3	CD8^+^	Total	−1.68
3	CD3	T cell	Central memory	−1.59
4	CD3	CD4^+^	Central memory	−1.58
5	CD28	CD8^+^	Total	1.58
6	CD3	CD4^+^	Total	−1.54
7	CD3	CD4	Effector memory	−1.53
8	CD3	CD8	Naïve	−1.52
9	CD3	T cell	Effector memory	−1.51
10	CD28	T cell	Total	1.49
11	CD3	CD8^+^	Effector memory	−1.48
12	CD3	Double positive	Effector memory	−1.47
13	CD3	Double negative	Total	−1.46
14	CD3	T cell	Naïve	−1.45
15	CD3	CD8^+^	Central memory	−1.45
16	CD3	Double negative	Effector memory	−1.42
17	CD28	Double positive	Naïve	1.39
18	CD3	Double negative	Naïve	−1.33
19	CD28	Double positive	Central memory	1.32
20	CD3	CD4^+^	Naïve	−1.32
21	CD28	CD4^+^	Total	1.30
22	CD28	CD4^+^	Naïve	1.28
23	CD3	Double positive	Total	−1.28
24	CD8	Double positive	Effector memory	−1.27
25	CD3	Double positive	Central memory	−1.25

Ninety one variables including variables from the combined expression profiles of CD3, CD4, CD8, CD25, CD28 and CD95 were analysed. The most discriminatory component was found to be principal component 2 (PC2). The percentage of variance accounted for by PC2 is shown between parentheses. Shown here are the top contributing variables to PC2.

### Cytokine secretion after *in vitro* stimulation

**Figure 3:**
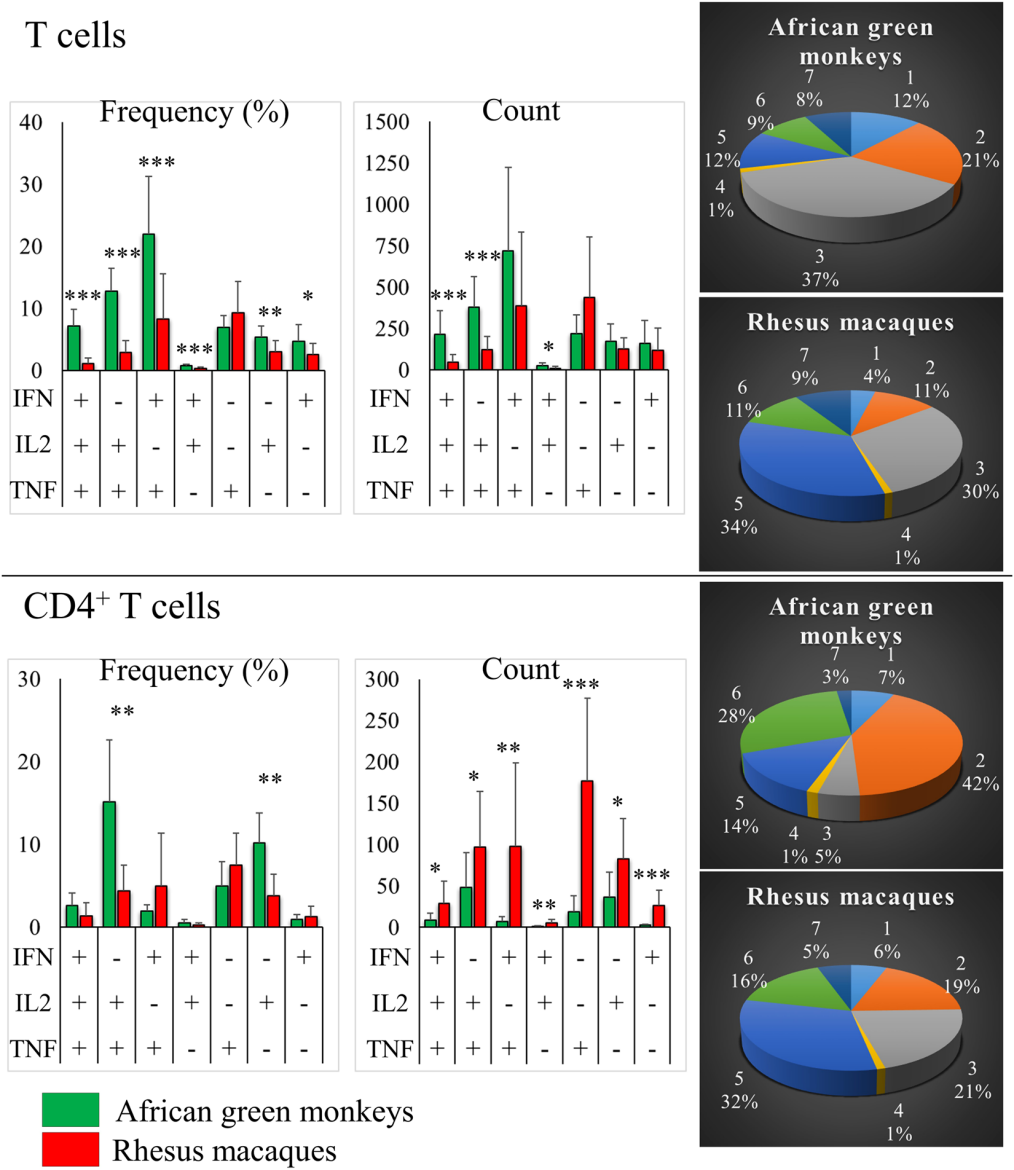
Cytokine-secretion patterns by African green monkey (n = 8) and rhesus macaque (n = 19) T cells. Cells were stimulated using phorbol 12-myristate 13-acetate and ionomycin for 6 hours, brefeldin A was added to inhibit protein secretion and cells were fixed and stained for the presence of cytokines as described in the methods section. Bars represent the arithmetic mean and error bars represent the standard deviation. Total T cell data (top) and CD4 + T cell data (bottom) are shown. Percentage (left bar charts and pie charts) and absolute count (middle bar charts) data were normalized by subtracting the corresponding data of unstimulated cells. Statistical significance was determined using a t-test and is indicated by “*”, “**” and “***” for 0.005 < *p* ≤ 0.05, 0.0005 < *p* ≤ 0.005 and 0.005 < *p* ≤ 0.0005, respectively. Pie slices 1 through 7 represent IFN^+^IL2^+^TNF^+^, IFN^−^IL2^+^TNF^+^, IFN^+^IL2^−^TNF^+^, IFN^+^IL2^+^TNF^−^, IFN^−^IL2^−^TNF^+^, IFN^−^IL2^+^TNF^−^ and IFN^+^IL2^−^TNF^−^ cell populations, respectively. Representative dot plots are shown in Supplemental Fig. S5.

**Figure 4:**
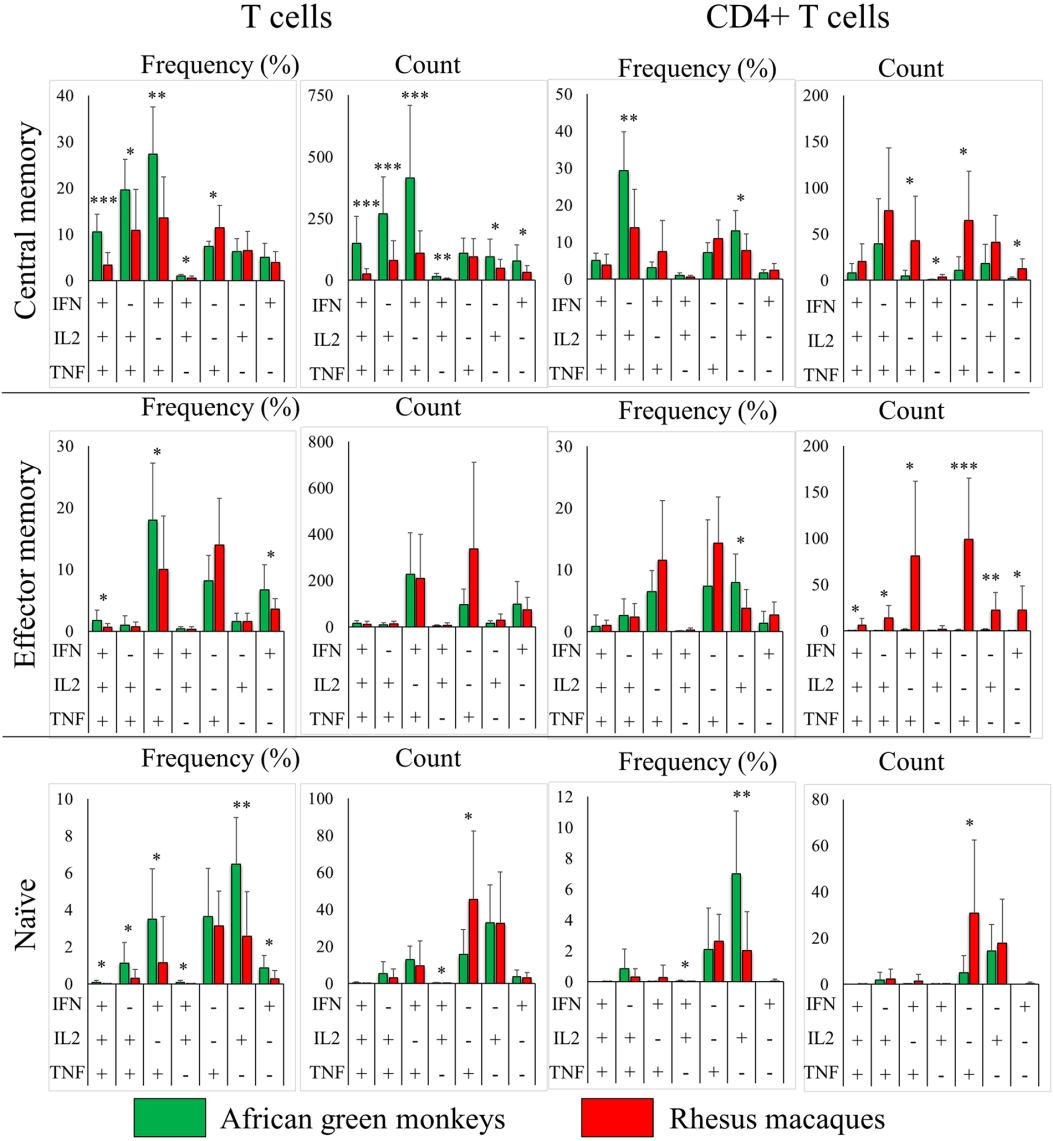
Cytokine-secretion patterns by African green monkey (n = 8) and rhesus macaque (n = 19) T-cell subsets. Cells were stimulated using phorbol 12-myristate 13-acetate and ionomycin for 6 hours, brefeldin A was added to inhibit protein secretion and cells were fixed and stained for the presence of cytokines as described in the methods section. Percentage (left) and absolute count (right) data were normalized by subtracting the corresponding data of unstimulated cells. Significantly different cell populations are indicated in the bar graphs by red plus signs (+). Each of the seven cell populations shown is depicted in both the bar graphs pie charts. Bars and pie slices representing the same population of cells have matching colours (i.e. pie splices and corresponding squares underneath each bar graph are colour-matched). Cells that secrete interferon gamma (red), interleukin 2 (green) or tumour necrosis factor alpha (blue) from all cell populations are indicated by coloured arcs around each pie chart. Representative dot plots and pie charts are shown in Supplemental Figs S5 and S6, respectively.

**Figure 5:**
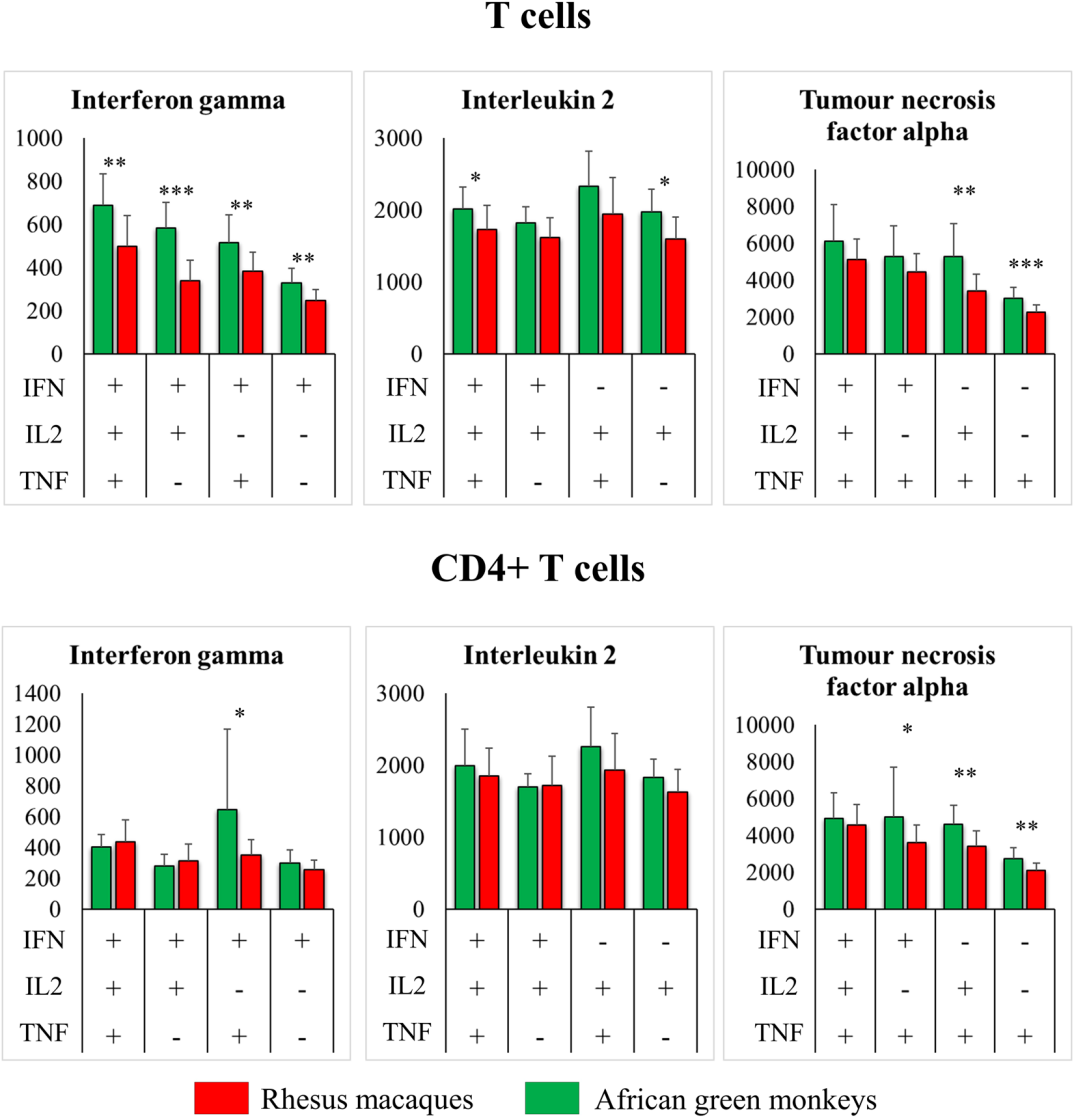
Quantitative cytokine-secretion patterns by African green monkey (n = 8) and rhesus macaque (n = 19) T cells. Cells were stimulated using phorbol 12-myristate 13-acetate and ionomycin for 6 hours, brefeldin A was added to inhibit protein secretion and cells were fixed and stained for the presence of cytokines as described in the methods section. Mean fluorescence intensity (MFI) was used as a quantitative measure of average cytokine production per cell. Significantly different cell populations are indicated by red plus signs (+). Representative dot plots are shown in Supplemental Fig. S5.

Historically, secretion of IL-2, IFN-γ and TNF-α has been used to evaluate T cell proinflammatory function^8^. Recent work has shown a correlation between *in vitro* cytokine secretion in response to chemical stimulants and protection^9^. Thus, we decided to stimulate AGM and RM T cells *in vitro* and compare their IL-2, IFN-γ and TNF-α secretion patterns. One of the most prominent differences found was the markedly higher abundance of multiple-cytokine producing cells among AGM T cells as a whole and in the central memory compartment compared to RMs. This difference was not observed in the T cell effector memory compartment (Figs 3 and 4). More specifically, central memory T cells that secreted two (double producers) or all (triple producers) of the three cytokines tested were more abundant in AGMs than in RMs. In the effector memory compartment, cells that only secreted TNF-α were less abundant in AGMs compared to RMs (Fig. 4). Among CD4^+^ T cells, those cells that secreted IL-2, alone in central and effector memory or in combination with TNF-α in central memory, were of higher percentage in AGMs. However, it is noteworthy that, as we and others^10,11^ have shown, CD4^+^ T cell counts are notably lower in AGMs than in RMs. Not surprisingly, we found fewer cytokine-secreting CD4^+^ T cells per cubic millilitre of blood in AGMs compared to macaques (Figs 3 and 4). In the naïve T-cell subset, including CD4^+^ T cells, the main difference between AGMs and RMs was the higher percentage of cells that secreted IL-2, but not TNF-α or IFN-γ, in the former compared to the latter (Fig. 4). In addition to testing the abundance of cytokine-secreting cells, we also wanted to compare average cytokine expression levels by AGM and RM T cells. For this purpose, MFI was used. Since others had reported higher cytokine MFI in multifunctional cells (i.e. cells that exhibit more than one function, such as cytokines and degranulation markers), we tested MFI in triple, double and single producer populations separately. We show that compared to RMs, AGMs showed higher IFN-γ levels in T cells, excluding CD4^+^ T cells; higher IL-2 levels in triple and single producer T cells, also excluding CD4^+^ T cells; and higher TNF-α levels in single producers and IL-2^+^ double producers in both CD4^+^ T cells and T cells as a whole (Fig. 5).

### Profiling cytokine-secretion patterns

**Figure 6:**
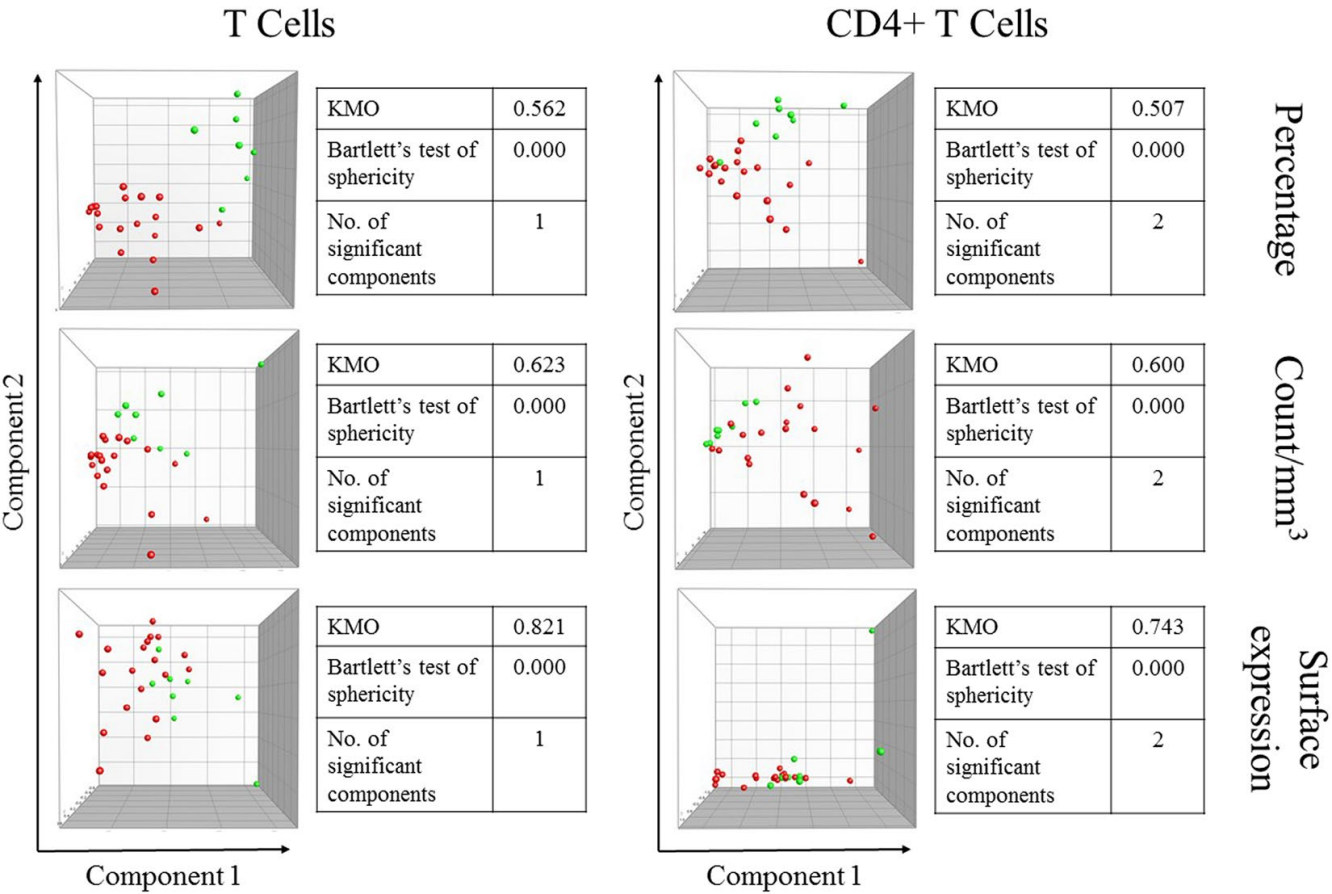
Segregation of African green monkeys (green; n = 8) and rhesus macaques (red; n = 19) based on secretion patterns of IFN-γ, IL-2 and TNF-α by overall T cells (left) and CD4 + T cells (right). Kaiser-Meyer-Olkin measure of sampling adequacy (KMO) and Bartlett’s test of sphericity p values are indicated. The number of significant components was determined using Monte Carlo simulation. Representative dot plots are shown in Supplemental Fig. S5.

Three cytokine secretion profiles were created for both total T cells and CD4^+^ T cells: percent, absolute count and mean expression profiles. Percent and absolute count profiles were composed of data for overall producers (i.e. percentage or absolute count of all cells that secreted a given cytokine regardless of whether they secreted any of the other two), single-cytokine producers (i.e. cells that secreted one of the three cytokines tested, but not the other two), double producers (i.e. cells that secreted two out of the three cytokines, but not the third) and triple producers (i.e. cells that secreted all three cytokines). Mean expression profiles were made of mean fluorescence intensity data for each cytokine in every population of cells that secreted it. Our data showed that AGMs and RMs were segregated best based on the percentage profile of total T cells, followed by that of CD4^+^ T cells (Fig. 6). Ranking variables by their contribution to the most discriminatory components showed that the overall percentages of cytokine secreting cells were most important, although all variables contributed substantially. Among absolute count variables, IFN-γ/TNF-α, and IL-2/TNF-α double producer, overall IFN-γ producer, IL-2 single producer and overall IL-2 producer cells were the top contributors to the most discriminatory component (Table 3).

**Table 3:** Variable contributions to the top principal components in principal component analysis of African green monkey (n = 8) and rhesus macaque (n = 19) cytokine production patterns.

Total T cells			Total CD4^+^ T cells		
Rank	Percent variable	PC1 (86%)	Rank	Percent variable	PC2 (11%)
1	TNF-α^+^	5.13	1	IFN-γ^+^ IL-2^−^ TNF-α^+^	−2.99
2	IFN-γ^+^	5.05	2	IFN-γ^−^ IL-2^+^ TNF-α^+^	2.21
3	IL-2^+^	4.99	3	IFN-γ^+^	−2.11
4	IFN-γ^+^ IL-2^−^ TNF-α^+^	4.93	4	IFN-γ^−^ IL-2^+^ TNF-α^−^	1.97
5	IFN-γ^−^ IL-2^+^ TNF-α^+^	4.86	5	IL-2^+^	1.91
6	IFN-γ^+^ IL-2^+^ TNF-α^−^	4.82	6	IFN-γ^+^ IL-2^−^ TNF-α^−^	−1.77
7	IFN-γ^−^ IL-2^+^ TNF-α^−^	4.78	7	IFN-γ^+^ IL-2^+^ TNF-α^−^	0.98
8	IFN-γ^+^ IL-2^+^ TNF-α^+^	4.71	8	IFN-γ^−^ IL-2^−^ TNF-α^+^	−0.65
9	IFN-γ^+^ IL-2^−^ TNF-α^−^	4.70	9	IFN-γ^+^ IL-2^+^ TNF-α^+^	0.16
10	IFN-γ^−^ IL-2^−^ TNF-α^+^	4.09	10	TNF-α^+^	−0.11

### IFN-γ-secreting CD4^+^ T cells display modest amounts of CD3 on their surfaces

**Figure 7:**
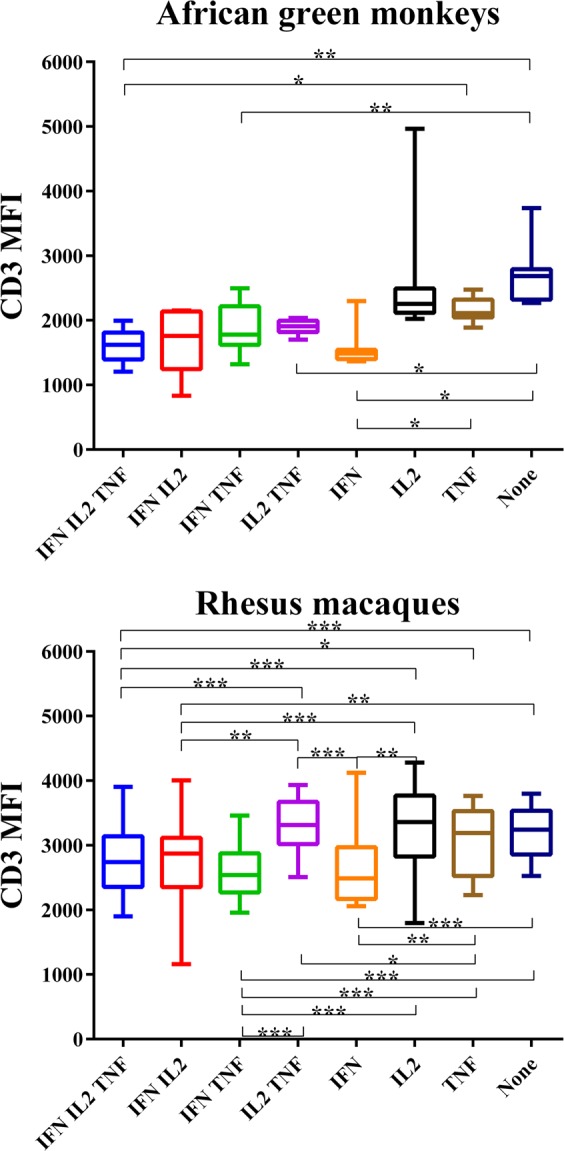
Differential surface expression of CD3 by central memory CD4 + T cells exhibiting different cytokine-secretion patterns after mitogenic stimulation in African green monkeys (top; n = 8) and rhesus macaques (bottom; n = 19). The top and bottom borders of each box denote the 75^th^ and 25^th^ percentiles, respectively. Middle lines denote medians. Whiskers denote maximum and minimum values. IFN IL2 TNF (blue bars): triple positive cells secreting interferon gamma (IFN-γ), interleukin 2 (IL-2) and tumour necrosis factor alpha (TNF-α); IFN IL2 (red bars): double positive cells secreting IFN-γ and IL-2; IFN TNF (green bars): double positive cells secreting IFN-γ and TNF-α; IL2 TNF (purple bars): double positive cells secreting IL-2 and TNF-α; IFN (orange bars): single positive cells secreting IFN-γ; IL2 (black bars): single positive cells secreting IL-2; TNF (gold bars): single positive cells secreting TNF-α; None (dark blue bars): triple negative cells. A two-tailed t-test was performed to evaluate the statistical significance in pairwise comparisons. “*”, “**” and “***” represent *p* values of 0.001 < *p* ≤ 0.01, 0.0001 < *p* ≤ 0.001 and *p* ≤ 0.0001, respectively. Significant differences with *p* values > 0.01, but < 0.05 that are not shown here are shown in Supplemental Fig. 8.

Our initial thought was: if lower CD3 surface expression is a marker of better SIV control, then multifunctional T cells — known for their robust immune responses and shown here to be more abundant in AGMs than in RMs — may express lower levels of surface CD3 compared to other cells. Therefore, we compared CD3 levels across different populations of central memory CD4^+^ T cells (see Supplemental Fig. S7 for representative dot plots). Central memory cells were chosen for their more robust responses than naïve cells and higher abundance compared to effector memory cells. Three main findings were identified. First, in both AGMs and RMs, IFN-γ-secreting cell populations showed lower levels of CD3 on their surface than IFN-γ-negative cells. These cells included triple producers and IFN-γ-secreting double producers, which showed lower levels of CD3 than IL-2-secreting and TNF-α-secreting single producers and triple negative cells. Secondly, in AGMs, all three populations of double producers showed equivalent levels of CD3 surface expression. In RMs, however, IFN-γ-secreting double producers showed lower levels of CD3 compared to IL-2^+^TNF-α^+^ cells. Finally, IL-2-secreting single producers and triple negative cells showed the highest levels of CD3 surface expression in both AGMs and RMs. The only possible exception to this observation is RMs’ TNF-α single producers, which expressed slightly lower levels of CD3 than triple negative cells, with the difference narrowly falling short of statistical significance (*p* = 0.0547) (Fig. 7, Supplemental Fig. S8). Taken together, our results suggest that a T cell’s ability to secret IFN-γ is a better predictor of low CD3 surface expression than multifunctionality, while IL-2 secretion predicts high levels of CD3 surface expression.

### Protective SIV challenge results in more multifunctional responses than pathogenic challenge in cynomolgus macaques (CMs)

**Figure 8:**
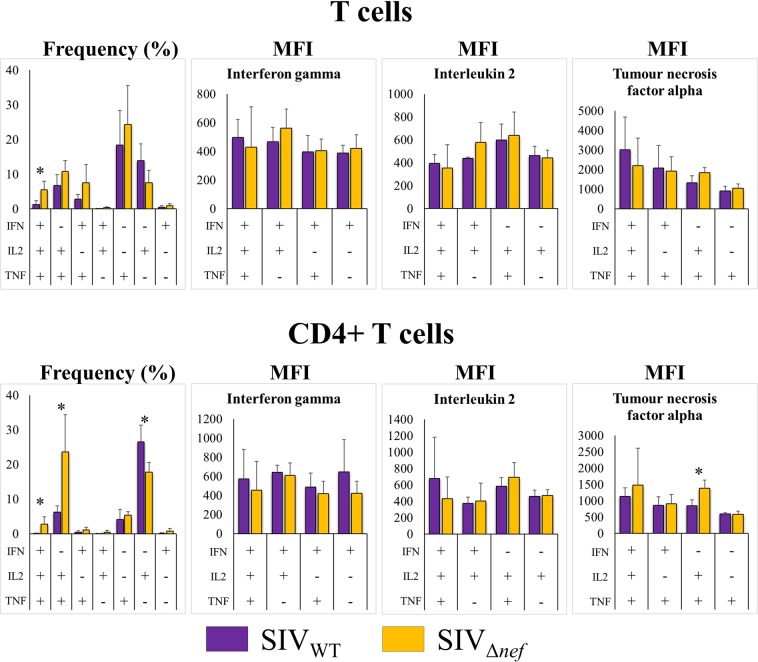
T-lymphocyte cytokine-secretion patterns after *in vitro* mitogen (PMA and ionomycin) stimulation of T cells from cynomolgus macaques infected with either wild-type or attenuated SIV (n = 4 each). Frequency (y axes): percentage of cells in parental population; MFI (y axes): mean fluorescence intensity; IFN: gamma interferon; IL2: interleukin 2; TNF: tumour necrosis factor alpha.

We do not know whether the higher abundance of multifunctional T cells in AGMs is causally linked to protection from AIDS. We thought it would be consistent with this notion, however, if the same phenomenon could be demonstrated in monkeys that had developed protective immunity, such as that developed in response to non-pathogenic SIV infection. Therefore, we asked whether non-pathogenic SIV infection augments multifunctional responses in susceptible animal species. We used CMs to answer this question. It has been previously shown that CMs vaccinated with replication-deficient viruses, such as SIV_mac239Δ*nef*_, develop protection against future challenge with wild-type viruses^12^. Unvaccinated CMs develop an AIDS-like syndrome that mimics human AIDS even more closely than the syndrome developed by RMs after pathogenic SIV infection^12^. Therefore, we decided to test whether CM T cells would produce higher abundance of multifunctional T cells after mitogenic stimulation than animals infected with the corresponding wild-type virus (SIV_mac239_). We found that, compared to animals infected with wild-type virus, SIV_mac239 Δ*nef*_-infected CMs had significantly higher percentage of triple producer T cells, which was true in both total T cells and CD4^+^ T cells, and IL-2/TNF-α-secreting CD4^+^ T cells, but lower percentage of IL-2 single producer CD4^+^ T cells. IL-2/TNF-α-secreting CD4^+^ T cells showed higher levels of TNF-α in SIV_mac239 Δ*nef*_-infected animals than the corresponding cell population in wild-type SIV_mac239_-infected animals. No other differences in average per-cell cytokine production were observed between animals infected with wild-type versus replication-defective SIV (Fig. 8).

## Discussion

The first objective in this study was to develop a multivariate approach to enable the study of complex immunological questions. Unbiased partitioning of data points was achieved using PCA. Bartlett’s test of sphericity, KMO and Monte Carlo simulation were used to verify the presence of correlated variables (a prerequisite for using PCA), adequacy of sample size and significance of PCs, respectively. One of the issues we encountered was the low or indeterminate KMO and the inability to calculate Bartlett’s test of sphericity for a few datasets. From a purely mathematical standpoint, KMO lower than 0.5 indicates that partial correlations are larger than correlations between variables, making PCA and other factoring methods unfit for the dataset. Optimally, such problems can be overcome by removing problematic variables or increasing sample size. In this study, we analysed the data using MDS and hierarchical clustering whenever good partitioning of AGMs and RMs was observed using PCA. Interestingly, the results obtained using MDS and hierarchical clustering supported PCA results, which increased our confidence in these results. In addition, the complete segregation of the two species obtained using T-cell population variables or combined surface molecules data is unlikely to have occurred due to random chance alone using all three analysis methods. Having said so, it is undeniable that studies utilizing larger sample sizes are warranted to substantiate the findings we present in this study.

The second objective was to apply our multivariate approach to identify the most discriminatory T-cell-related variables that best distinguish between AGMs and RMs. We selected AGMs and RMs for the former’s ability to survive SIV infections, and the latter’s mimicry of human lentiviral infections. Primate lentiviruses replicate efficiently in their African natural hosts (e.g. AGMs and sooty mangabeys), yet these infections are almost always non-pathogenic^13–17^. On the contrary, untreated lentiviral infections are generally fatal in humans, RMs and CMs^12,17–21^. This striking difference in infection outcome with the same or very similar viruses inspired many studies that aimed to determine the differences in lentiviral-specific immune responses between AGMs and other SIV natural hosts on the one hand and humans and macaques on the other hand.

Although a complete understanding of the mechanisms underlying non-pathogenic infections in natural hosts remains elusive, multiple studies were able to propose specific mechanisms and reach a consensus on a set of clear phenotypes that separate natural hosts from humans and RMs. The most prominent among these phenotypes are (1) the absence of chronic immune activation during the chronic phase of infection^17,22^, (2) paucity of total CD4^+^ and CD4^+^CCR5^+^ T cells^11,23^, (3) down regulation of surface CD4 expression by memory cells^10,11^, (4) low levels of CCR5 surface expression by memory CD4^+^ T cells^23,24^ and (5) the presence of functional T-helper CD3^+^CD4^−^CD8^−^ cells in natural hosts^10,11^. It has been suggested that viral replication in natural hosts might be restricted by the limited pool of susceptible cells^17^ due to down regulation of the primary viral receptor (CD4)^10,11^ and a major co-receptor (CCR5)^23,24^. However, the actual contribution of this mechanism *in vivo* is not clear, since natural SIV hosts experience high viremia that is often comparable to that experienced by experimentally infected RMs^17^. Our current knowledge in this area is based almost exclusively on post-infection, univariate studies. Pre-infection differences and multivariate profiling remain largely unexplored. It is not doubtful, however, that pre-existing differences between AGMs and RMs play a prominent role in shaping the immune response upon antigen encounter. For this reason, we decided to apply our multivariate approach to study uninfected animals.

Since it was not possible to study all types of immune cells, we focused on T cells, particularly CD4^+^ T cells. Since our goal was to activate as many T cells as possible, we stimulated the cells chemically using a combination of PMA and ionomycin. PMA stimulates T cells by directly activating protein kinase C^25,26^, while ionomycin mobilizes calcium from intracellular stores and also activates protein kinase C^27^. As such, these chemical stimulants bypass the T-cell receptor (TCR) and activate most cells regardless of their antigen specificity. We were particularly interested in this mechanism of activation since it triggers signalling pathways directly and is, thus, not influenced by the affinity of antigen interaction with the TCR. Another option to stimulate large numbers of T cells is by using a superantigen. Although superantigen stimulation bears closer resemblance to physiological conditions, our experiments using staphylococcal enterotoxin B (a known superantigen^28,29^) could not achieve the magnitude or breadth of stimulation achieved by PMA/ionomycin (data not shown). Therefore, we made our choice to use the latter. Similarly, the variables selected for inclusion in this study included commonly measured variables with known physiological significance, such as cytokines and surface molecules. We realize that limiting our study to these variables and cell types was an unescapable limitation, and other investigations involving other cell types, variables, and stimulation regimens are likely equally meritorious.

The most biologically relevant findings we present here include the findings that CD3 and CD28 are the best discriminatory surface molecules between AGMs and RMs, and that AGM T cells tend to become multifunctional more readily than RM T cells. The CD3 molecule forms a complex with the TCR and acts as the signalling subunit responsible for initiating a signalling cascade leading to T cell activation and proliferation upon antigen encounter^30^. In addition to TCR ligation, a co-stimulatory signal is required for T cells to survive and proliferate. In the absence of co-stimulation, T cells become anergic or undergo apoptosis. CD28 is the most extensively studied and most effective in delivering a co-stimulatory signal in T cells^31^. We found lower levels of CD3 expressed on the surface of AGM T cells in the resting state and higher CD28 levels in the activated state to be among the most distinctive features separating these animals from RMs. We showed disparities in CD3 surface expression among physiologically distinct subsets of CD4^+^ T cells, suggesting that the levels of CD3 on the cell surface may play a role in shaping the quality of T cell responses. Our findings are consistent with a recent study suggesting that higher CD3 levels may augment T cell activation capacity^32^, conceivably by promoting serial binding to peptide:MHC complexes^32,33^. High surface expression of CD3 might become detrimental in lentiviral infections if it leads to persistent immune activation, although we do not have evidence linking CD3 levels to disease progression in RMs, or linking low CD3 expression to survival of AGMs through lentiviral infections. CD28 signalling has been shown to boost cytokine production and promote Th2 responses^31^. We show higher levels of specific cytokines in AGMs compared to RMs, but some other cytokines are actually higher in RMs. Furthermore, we only tested proinflammatory (Th1) cytokines, leaving the physiological implications of higher CD28 expression in AGMs unclear. Although not addressed in the current study, possible links between multifunctionality and levels of CD3 and CD28 are of prime interest and we plan to address such possibilities in our future investigations. Specifically, the role of CD3 and CD28 in “tweaking” downstream signalling, and whether it relates to multifunctionality, is worthy of further explorations. Another intriguing finding is the higher abundance of double negative cells in AGMs compared to RMs (data not shown), which we found to be critical for distinguishing between the two species. Previous studies suggested that double negative cells may be key in the survival of natural hosts of SIV by providing a pool of T-helper cells that is resistant to infection^10,11,34,35^.

Another distinctive feature we found in AGMs is the higher abundance of multifunctional central memory T cells in comparison to RMs. Multifunctional cells have been shown to secrete higher levels of cytokines and correlate with protection in multiple infectious diseases^36,37^. We have also seen higher per-cell levels of cytokines — as determined by mean fluorescence intensity — in AGMs compared to RMs. The observed higher tendency to secrete multiple cytokines and higher per-cell cytokine levels may reflect more robust immune responses mounted by AGMs compared to macaques. This is consistent with the more robust antibody responses found in AGMs^38^, which require the T-helper function of T cells. This work was done on chemically-stimulated cells *in vitro*, however. It would be interesting to see how our results compare to natural stimulation *in vivo* or to *in vitro* stimulation through the TCR. Given the high levels of plasma viremia in SIV natural hosts, immunological robustness alone cannot explain the non-pathogenic nature of the infection^17,39^. Of particular interest is the fact that multifunctional cells were also found in CMs vaccinated with a defective virus, suggesting that those cells could be used as markers of immune competence. Clearly, distinct populations of multifunctional cells are, almost certainly, distinct physiologically. Considering the functions possessed by a particular cell — many of which we may not have measured — and its developmental stage, numerous cell types must be in existence. Taken together, it is quite evident that a complex combination of responses is at play, which highlights the need for a multivariate approach in future immunological studies.

## Methods

### Animals, samples and PBMC purification

Indian-origin RMs (*Macaca mulatta*) were housed at the Wisconsin National Primate Research Centre, University of Wisconsin – Madison (Madison, WI). African green (vervet) monkeys (*Chlorocebus pygerythrus*) were housed by Primate Biologicals. Animal care and sample collection were conducted with strict adherence to the guidelines of the appropriate Institutional Animal Care and Use Committee and to the NIH “Guide to the Care and Use of Laboratory Animals.” Since our laboratory was not involved in collecting blood samples, keeping or caring for animals, and was not involved in setting up or modifying protocols for any of these activities, it was determined by the University of Wisconsin – Milwaukee Office of Safety and Assurances, Animal Care Program that we did not need an approved animal care protocol. Our laboratory has obtained all other institutional approvals, training and certification in relation to biosafety and blood-borne pathogen universal precautions. Whole blood samples were collected from 20 RMs and 8 AGMs. Samples were collected in EDTA tubes to prevent coagulation. Peripheral blood polymorphonuclear cells (PBMC) were isolated by layering diluted blood on lymphocyte separation medium followed by gradient centrifugation as per manufacturer’s protocol. Archived PBMC that had been collected from SIV_mac239_- and SIV_mac239Δ*nef*_-infected CMs (*Macaca fascicularis*) were a gift from David H. O’Connor (University of Wisconsin – Madison) and Justin M. Greene (Oregon Health and Science University).

### *In vitro* stimulation

Cells were cultured in RPMI 1640 medium containing L-glutamine (2.05 mM), penicillin (100 IU/mL), streptomycin (100 μg/mL) and 10% heat-inactivated foetal bovine serum. Cells were stimulated by culturing in medium containing PMA (50 ng/mL) and ionomycin (500 ng/mL) for 6 hours at 37 °C in 5% carbon dioxide atmosphere. In experiments requiring detection of cytokines’ secretion, protein secretion was blocked using Brefeldin A (BD Biosciences, San Jose, CA) two hours after adding stimulants Brefeldin A was added to the final concentration recommended by the manufacturer.

### Flow cytometry

PBMC surface markers were stained using anti-CD3-peridinin chlorophyll protein (PerCP)-Cy5.5 (clone: SP34-2), anti-CD4-phycoerythrin (PE)-CF594 (clone: L200), anti-CD8-PE-Cy7 (clone: RPA-T8), anti-CD25-allophycocyanin (APC) (clone: M-A251), anti-CD28-fluorescein isothiocyanate (FITC) (clone: CD28.2) and anti-CD95-PE (clone: DX2). For cytokine staining, surface staining with anti-CD3-PerCP-Cy5.5, anti-CD4-PE-CF594, anti-CD45RA-PE-Cy5 (clone: 5H9) and anti-CD95-PE was followed by intracellular staining with anti-IL-2-FITC (clone: MQ1-17H12), anti-IFN-γ-APC (clone: 4 S.B3) and anti-TNF-α-PE-Cy7 (clone: MAb11). All antibodies were purchased from BD Biosciences (San Jose, CA). For surface marker studies, CD4^+^ and CD8^+^ T cells were classified as central memory, effector memory and naïve as described in the results section and in Supplemental Fig. S1. Cytokine studies were performed on total T cells, CD4^+^ T cells or a specific subset as described in the appropriate section. In the cytokine studies, central memory, effector memory and naïve compartments were defined as CD45RA^−^CD95^+^, CD45RA^−^CD95^+^ and CD45RA^+^CD95^−^, respectively. Dead cells were stained with violet live/dead fixable dead cell stain (Life Technologies, Grand Island, NY). Flow cytometry was done using BD FACSAria III and data were analysed using FlowJo software (FlowJo, LLC, Ashland, OR). Cytokine-secretion patterns were analysed using SPICE version 5.1 downloaded from http://exon.niaid.nih.gov40.

### Principal component analysis

PCA was performed using BioNumerics 6.6 (Applied Maths, Austin, TX) or IBM SPSS version 22 (IBM Corporation, Armonk, NY). Bartlett’s sphericity test^41^ and KMO^42,43^ were used to evaluate the appropriateness of applying PCA to various datasets. Bartlett’s sphericity test verifies the existence of correlated variables. Since PCA is a data reduction technique that reduces the number of variables by condensing correlated variables into principal components, it is only appropriate to use PCA in the presence of correlated variables. In the absence of any correlation between variables, those variables are described mathematically as orthogonal and their correlation matrix is described as an identity matrix (i.e. a matrix composed of “1”s in the diagonal and “0”s elsewhere). Therefore, the null hypothesis — that there is no correlation between any of the variables — is upheld whenever the variables’ correlation matrix is an identity matrix and is rejected whenever the possibility of equality to an identity matrix approaches zero. In the present study, Bartlett’s sphericity test was used to reject the null hypothesis that the correlation matrix is identical to the identity matrix and a p value ≤ 0.05 was accepted. KMO measure of sampling adequacy compares correlations to partial correlations between variables. Partial correlations result when the correlation between two correlated variables is influenced by a third variable. When partial correlations are high relative to correlations, KMO approaches zero and the use of PCA is inappropriate. On the contrary, KMO values approaching 1 indicate that partial correlations are very small compared to correlations between variables and, thus, the use of PCA is appropriate. In this study, KMO values of ≥0.5 were considered acceptable^42^. Both Bartlett’s sphericity test and KMO measure of sampling adequacy were done using IBM SPSS version 22. The number of statistically significant components to be extract when performing PCA was determined using Parallel Analysis — also known as Monte Carlo simulation — using Brian O’Connor’s syntax for SPSS^44^. In this study, 1000 permutations (datasets) and eigenvalues were computed for 50^th^ and 95^th^ percentiles. The eigenvalues obtained from PCA were compared to the eigenvalues generated from Monte Carlo simulation in scree plots. Components were considered statistically significant when they had higher eigenvalues compared to the corresponding simulated 95^th^ percentile values.

### Other statistical methods

MDS and hierarchical cluster analysis were done using BioNumerics. The input into either method is a similarity matrix. Similarity matrices were calculated using Canberra distances (equation 1). Dendrograms were constructed using Unweighted Pair Group Method with Arithmetic Mean. Statistical significance of differences between AGMs and RMs in the percentage, absolute count and mean fluorescence intensity variables was determined using multiple t-tests with Holm-Sidak correction for multiple comparisons, which was performed using GraphPad Prism 6 (GraphPad Software, Inc., La Jolla, CA) for variables other than Boolean data. Cytokine Boolean data were analysed using SPICE version 5.1^40^ where significance was determined using the two-tailed t-test.

1 $$D=\frac{1}{n}\,{\sum }_{i=1}^{n}\frac{|Xi-Yi|}{|Xi+Yi|}$$

## Supplementary information

Supplementary Information
