# Supplementary material for: Multivariate profiling of African green monkey and rhesus macaque T lymphocytes
Source: Sci Rep. 2019 Mar 18;9:4834. doi: 10.1038/s41598-019-41209-x (PMC6423277; doi:10.1038/s41598-019-41209-x)

Multivariate profiling of African green monkey and rhesus macaque T lymphocytes

Wail M. Hassan [1, 2]*

Gregory F. Burton [3]

Gabriella A. Pinter [4]

Istvan G. Lauko [4]

Nader N. Mahdi [2]

Mackenzie E. Johnson [2]

[1] Department of Biomedical Sciences, University of Missouri Kansas City School of Medicine, Kansas City, MO, USA

[2] Department of Biomedical Sciences, University of Wisconsin – Milwaukee, Milwaukee, WI, USA

[3] Department of Chemistry and Biochemistry, Brigham Young University, Provo, UT, USA

[4] Department of Mathematical Sciences, University of Wisconsin – Milwaukee, Milwaukee, WI, USA

* Corresponding author: University of Missouri Kansas City School of Medicine, 2411 Holmes Street, M3-417, Kansas City, MO 64108; hassanwm@umkc.edu; Tel: (816) 235-6478; Fax: (816) 235-5277

Figure S1. Gating strategy for the identification of T cell subsets. After exclusion of dead cells, T cells ($CD3^+$) were classified into $CD4^+CD8^–$, $CD4^–CD8^+$, $CD4^+CD8^+$ (double positive) and $CD4^–CD8^–$ (double negative). Each of these four categories was further divided into naïve ($CD28^{lo}CD95^–$), central memory (CM) ($CD28^{hi}CD95^+$) and effector memory (EM) ($CD28^–CD95^+$). The same analysis was applied to all African green monkey (n = 8) and rhesus macaque (n = 19) samples.

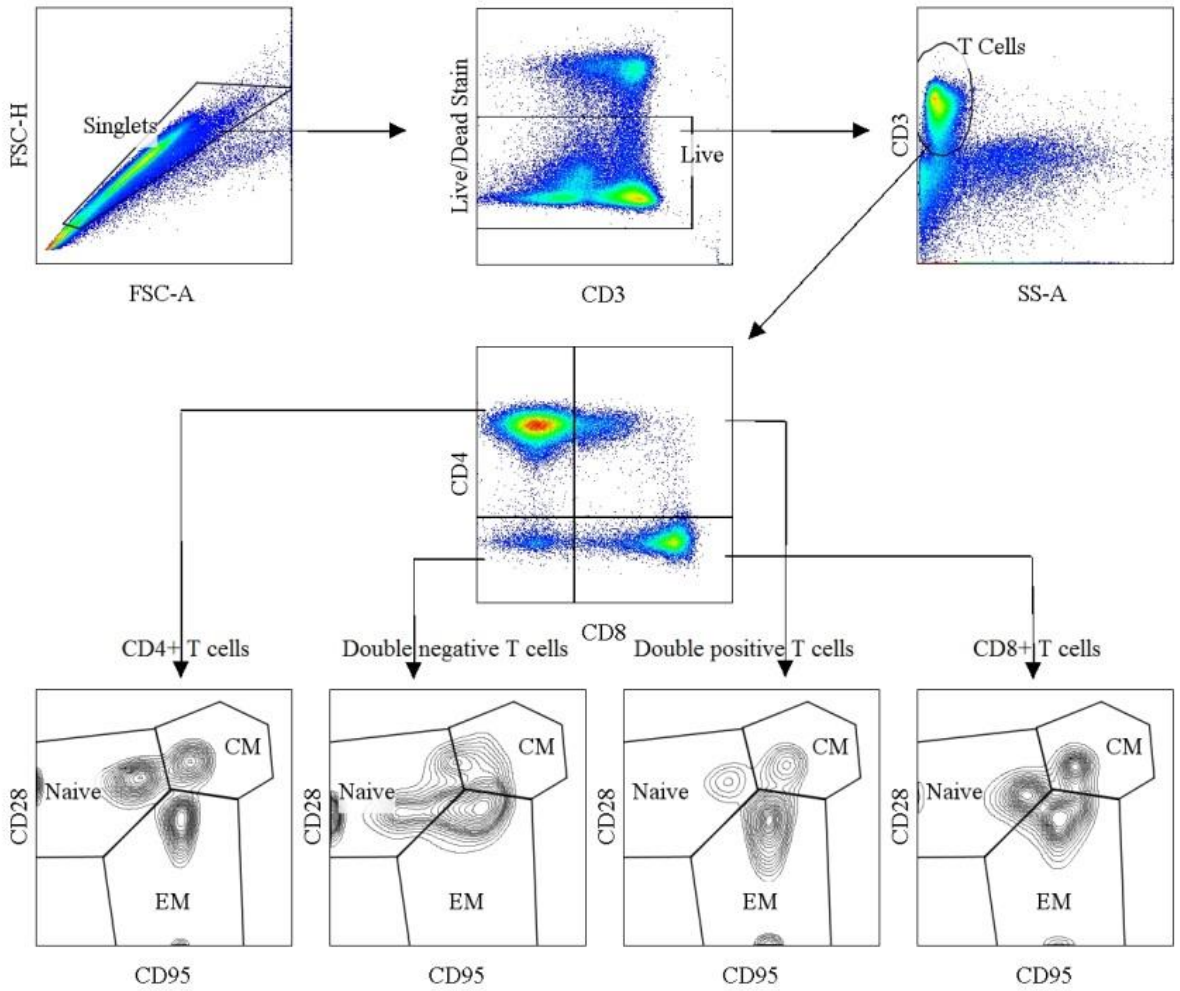

Figure S2. Hierarchical clustering and multidimensional scaling analysis of African green monkey (green; n = 8) and rhesus macaque (red; n = 19) T-cell subpopulations. Analysis based on percent (A, B) and absolute count (C, D) data are shown. Hierarchical clustering (A, C) and multidimensional scaling (B, D) were based on similarity calculated using Canberra distances (see Methods for description of similarity calculation).

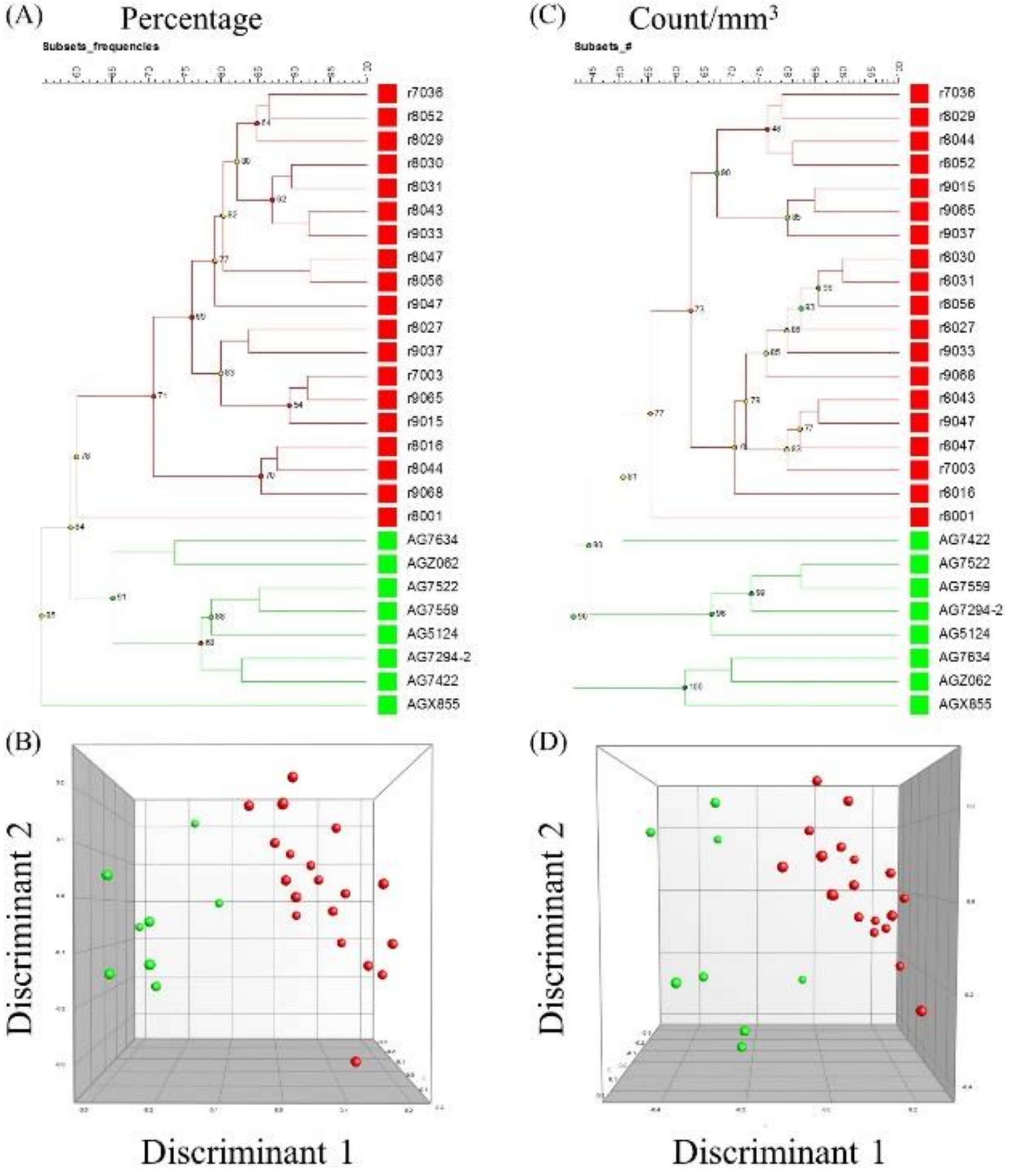

Figure S3. Representative dot plots showing surface-marker gating strategies. Shown in this figure are CD25, CD28 and CD95 gating in CD4^{+} and CD8^{+} T cells of African green monkeys (n = 8) and rhesus macaques (n = 19).

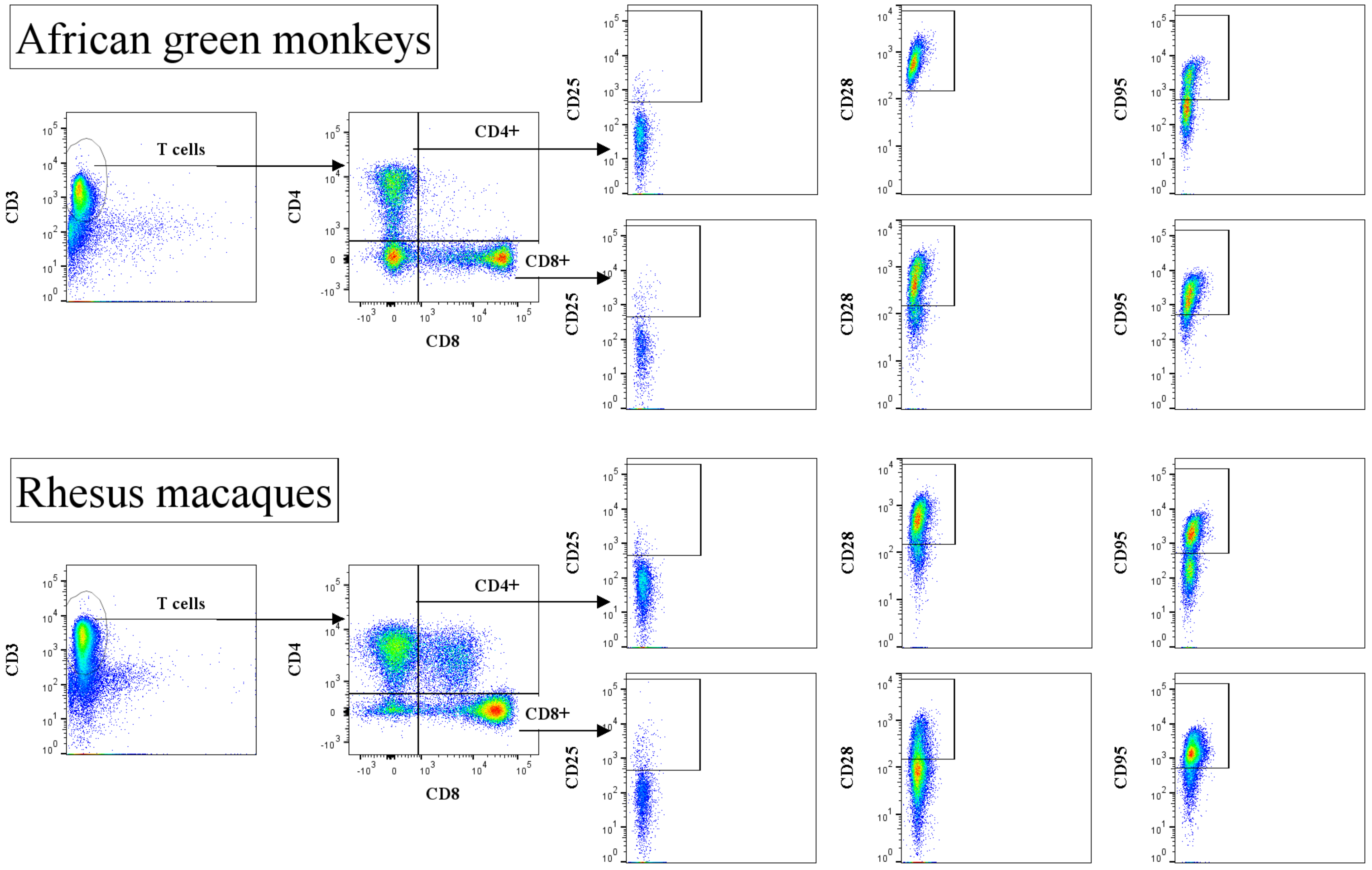

Figure S4. Segregation of African green monkeys (AGM; green; n = 8) and rhesus macaques (RM; red; n = 19) based on T-cell mean surface expression of CD3, CD4 (A-D), CD8 (E-H), CD25 (I-L), CD28 and CD95 (M-P). Data were analysed using principal component analysis (A, E, I, M, Q), multidimensional scaling (B, F, J, N, R) and hierarchical clustering (D, H, L, P, S) on individual surface proteins (A-P) or a combined profile of all six surface molecules (Q-S). To determine component significance in principal component analysis, scree plots showing raw eigenvalues and eigenvalues modelled at 50th and 95th percentile (blue, green and yellow, respectively) were used. Components were considered significant when their raw eigenvalues were higher than the corresponding 95th percentile modelled values. Kaiser-Meyer-Olkin measure of sampling adequacy (KMO), Bartlett's test of sphericity *p* values and the number of significant components are indicated below each plot. Surface expression levels of CD4, CD8, CD25 and CD95 as determined by mean fluorescence intensity of conjugated antibody stained cells are shown (C, G, K, O). Statistical significance of differences between African green monkeys and rhesus macaques in surface-molecules' expression was determined using multiple t-tests with Holm-Sidak correction for multiple comparisons. *P* values below 0.05 are indicated.

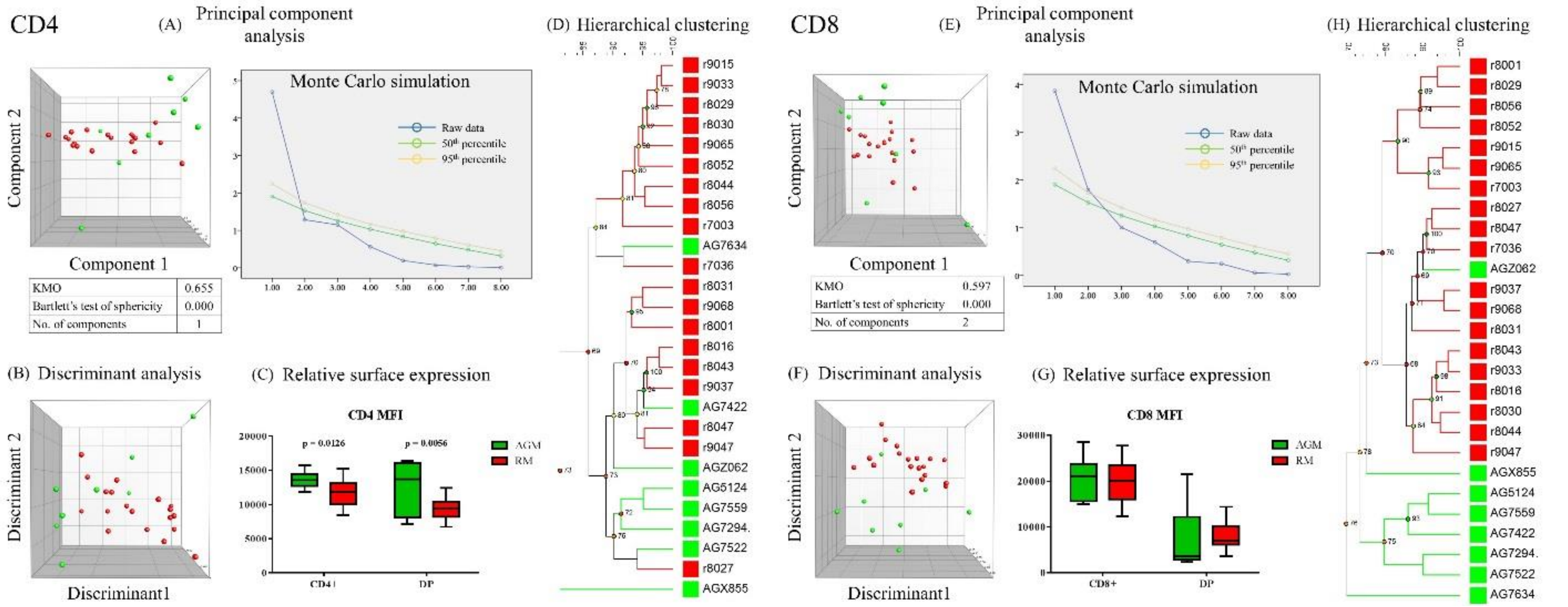

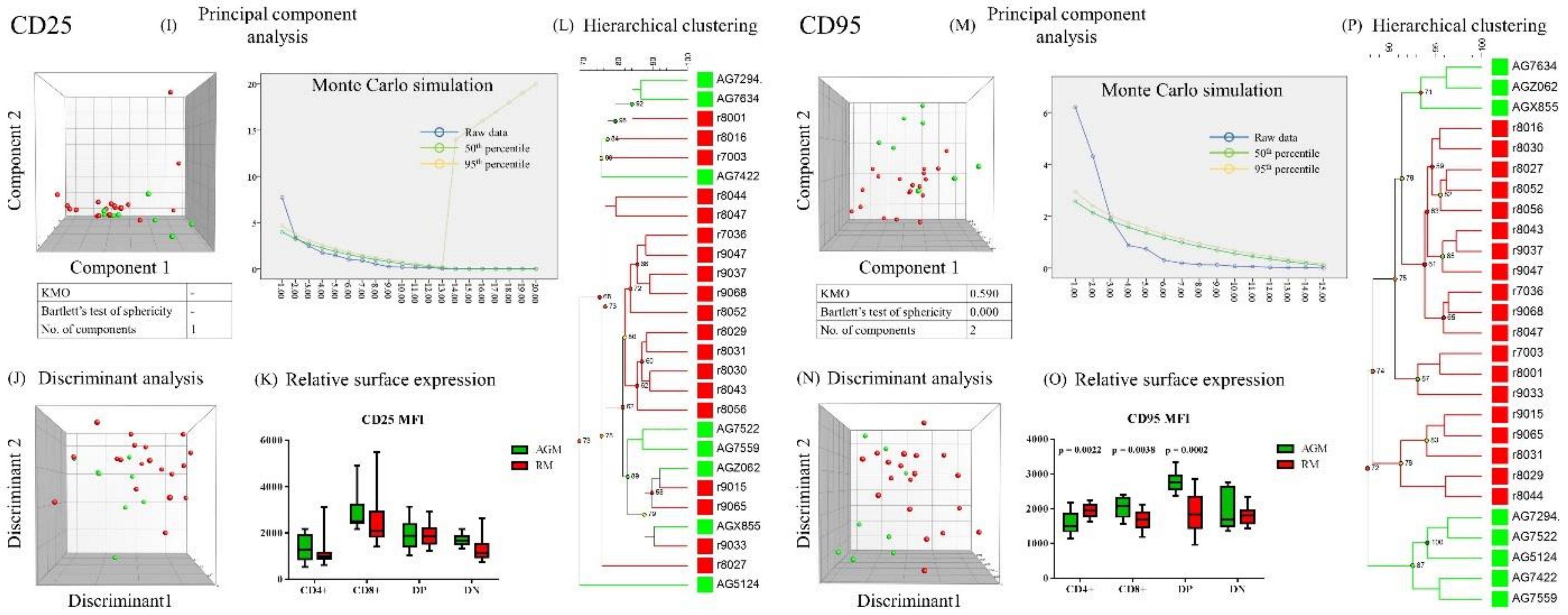
CD25
(I) Principal component analysis
Monte Carlo simulation
Raw data
50th percentile
95th percentile
Component 1
Component 2
KMO -
Bartlett's test of sphericity -
No. of components 1
(J) Discriminant analysis
Discriminant 1
Discriminant 2
(K) Relative surface expression
CD25 MFI
AGM
RM
CD4+
CD8+
DP
DN
(L) Hierarchical clustering
AG7294.
AG7634
r8001
r8016
r7003
AG7422
r8044
r8047
r7036
r9047
r9037
r9068
r8052
r8029
r8031
r8030
r8043
r8056
AG7522
AG7559
AGZ062
r9015
r9065
AGX855
r9033
r8027
AG5124
CD95
(M) Principal component analysis
Monte Carlo simulation
Raw data
50th percentile
95th percentile
Component 1
Component 2
KMO 0.590
Bartlett's test of sphericity 0.000
No. of components 2
(N) Discriminant analysis
Discriminant1
Discriminant 2
(O) Relative surface expression
CD95 MFI
p = 0.0022
p = 0.0038
p = 0.0002
AGM
RM
CD4+
CD8+
DP
DN
(P) Hierarchical clustering
AG7634
AGZ062
AGX855
r8016
r8030
r8027
r8052
r8056
r8043
r9037
r9047
r7036
r9068
r8047
r7003
r8001
r9033
r9015
r9065
r8031
r8029
r8044
AG7294.
AG7522
AG5124
AG7422
AG7559

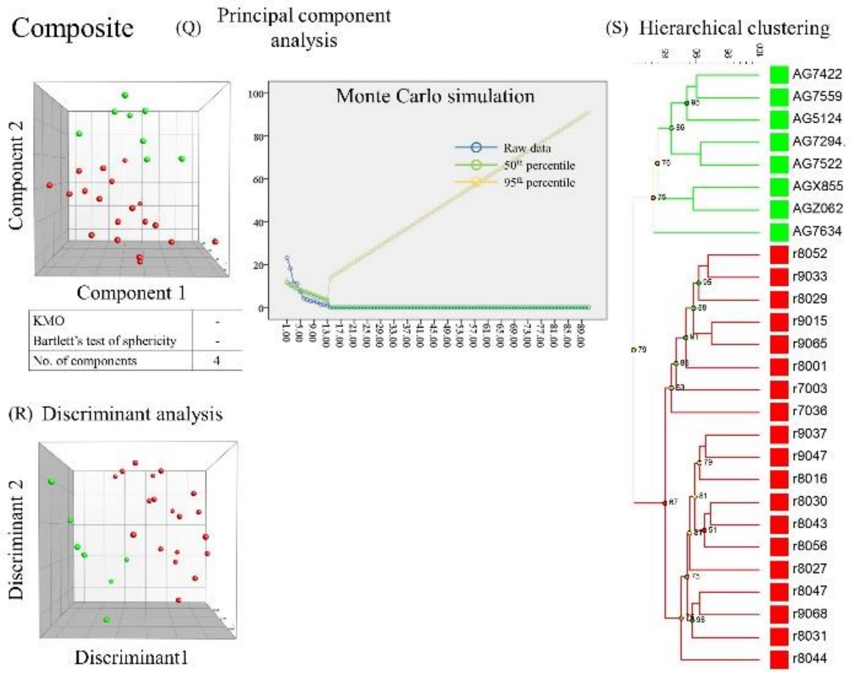
Composite
(Q) Principal component analysis
Component 2
Component 1
KMO -
Bartlett's test of sphericity -
No. of components 4
Monte Carlo simulation
Raw data
50th percentile
95th percentile
(R) Discriminant analysis
Discriminant 2
Discriminant1
(S) Hierarchical clustering
AG7422
AG7559
AG5124
AG7294
AG7522
AGX855
AGZ062
AG7634
r8052
r9033
r8029
r9015
r9065
r8001
r7003
r7036
r9037
r9047
r8016
r8030
r8043
r8056
r8027
r8047
r9068
r8031
r8044

Figure S5. Representative dot plots showing gating strategies used to identify cytokine-secreting total T cells and CD4$^+$ T cells of African green monkeys (n = 8) and rhesus macaques (n = 19). Dot plots: x axes show side scatter, y axes show brightness of cytokine staining in arbitrary units. Histograms: x axes show brightness of cytokine staining in arbitrary units, y axes show cell counts normalized to mode (%). Overlayed data of phorbol 12-myristate 13-acetate and ionomycin (PMA + I)-stimulated and unstimulated cells are shown in each histogram. IFNg: gamma interferon; IL-2: interleukin 2; TNFa: tumour necrosis factor alpha.

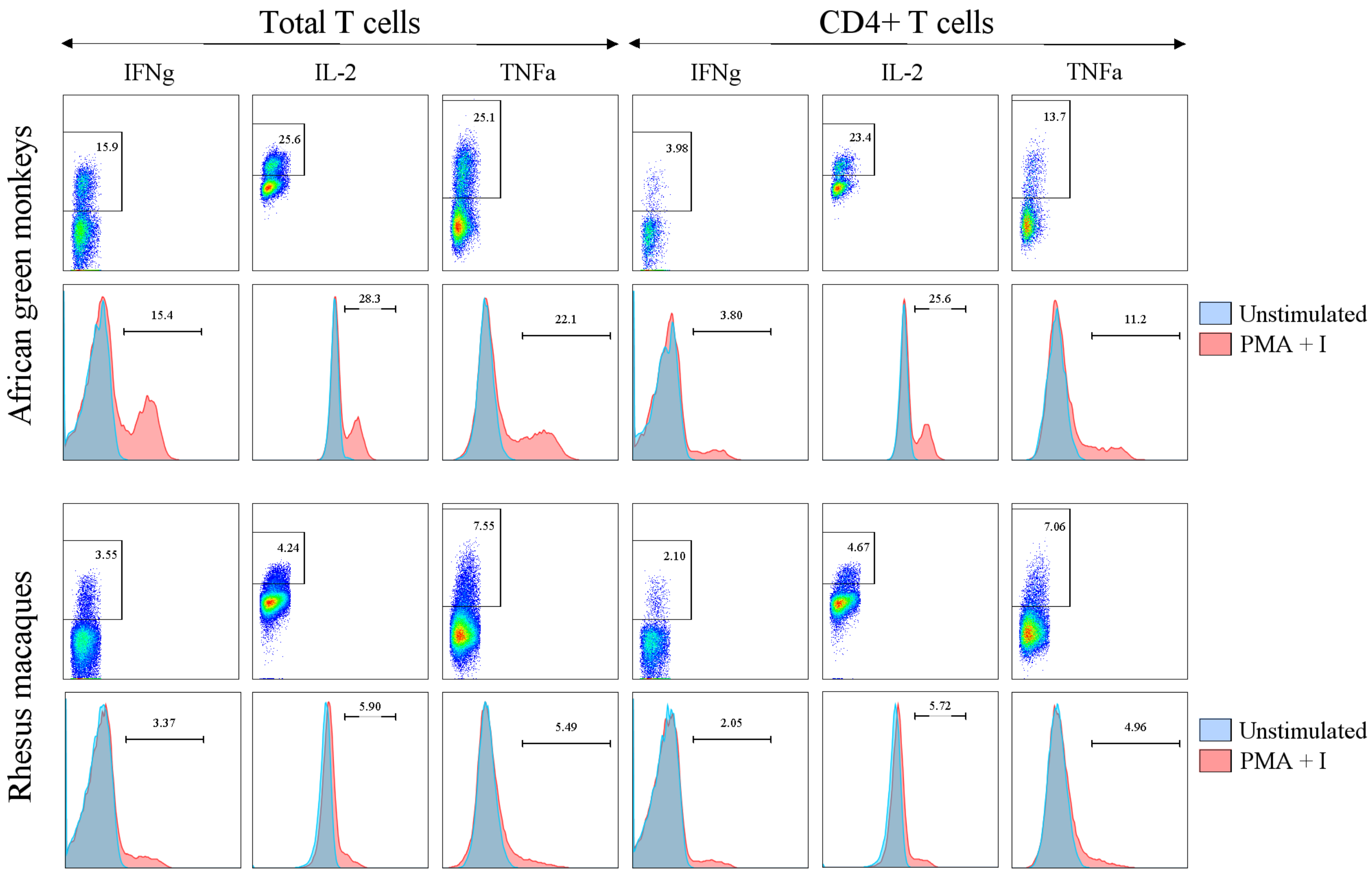

Figure S6. Cytokine-secretion patterns of the central memory, effector memory and naïve compartments of total T cells and CD4^{+} T cells of African green monkeys (n = 8) and rhesus macaques (n = 19). Pie slices 1 through 7 represent IFN^{+}IL2^{+}TNF^{+}, IFN^{-}IL2^{+}TNF^{+}, IFN^{+}IL2^{-}TNF^{+}, IFN^{+}IL2^{+}TNF^{-}, IFN^{-}IL2^{-}TNF^{+}, IFN^{-}IL2^{+}TNF^{-} and IFN^{+}IL2^{-}TNF^{-} cell populations, respectively.

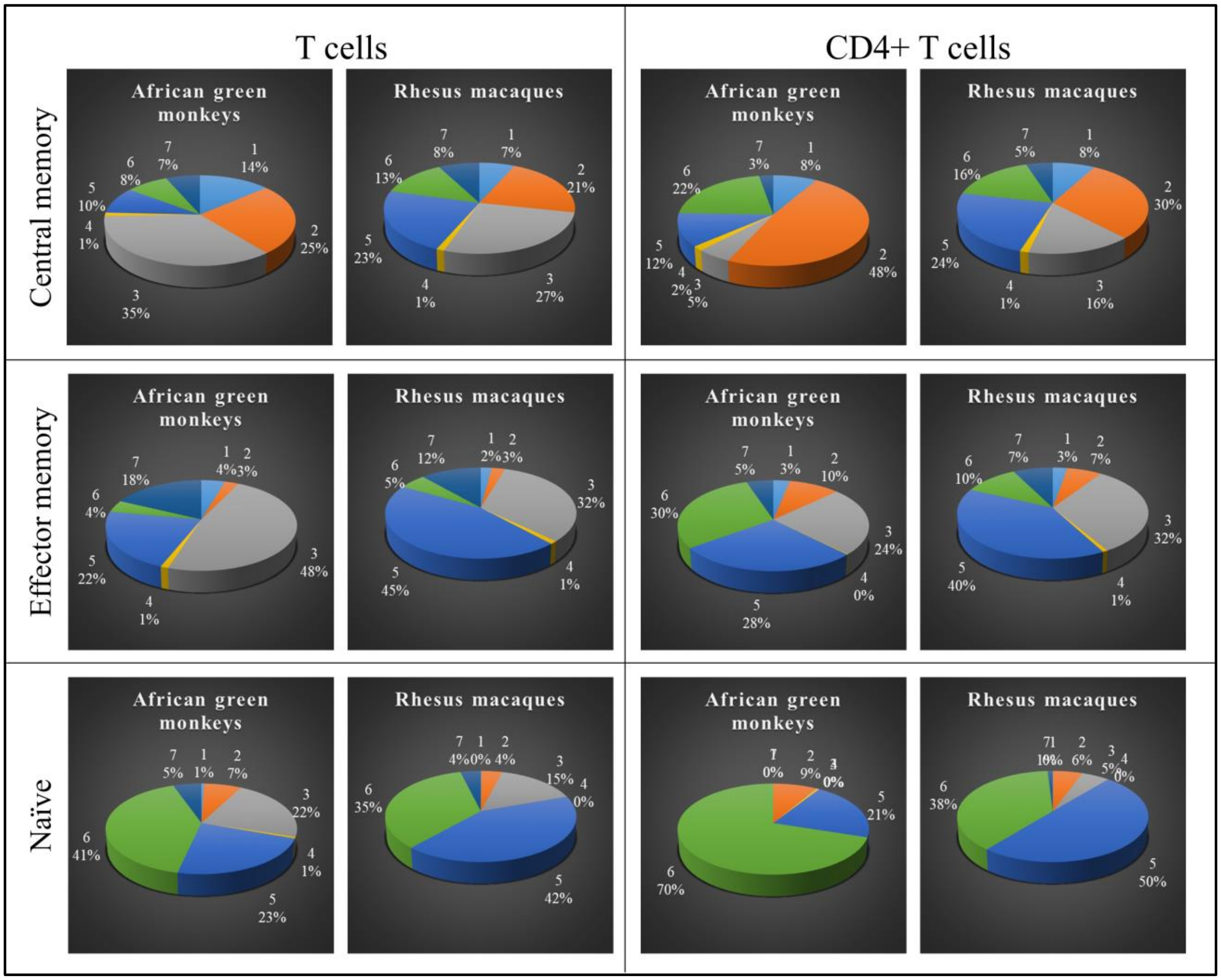

Figure S7. Representative dot plots showing gating on CD4+ central memory T cells of African green monkeys (n = 8) and rhesus macaques (n = 19). Cytokine secretion is shown in overlaid histograms from phorbol 12-myristate 13-acetate and ionomycin (PMA + I)-stimulated and unstimulated cells. IFNg: gamma interferon; IL-2: interleukin 2; TNFa: tumour necrosis factor alpha.

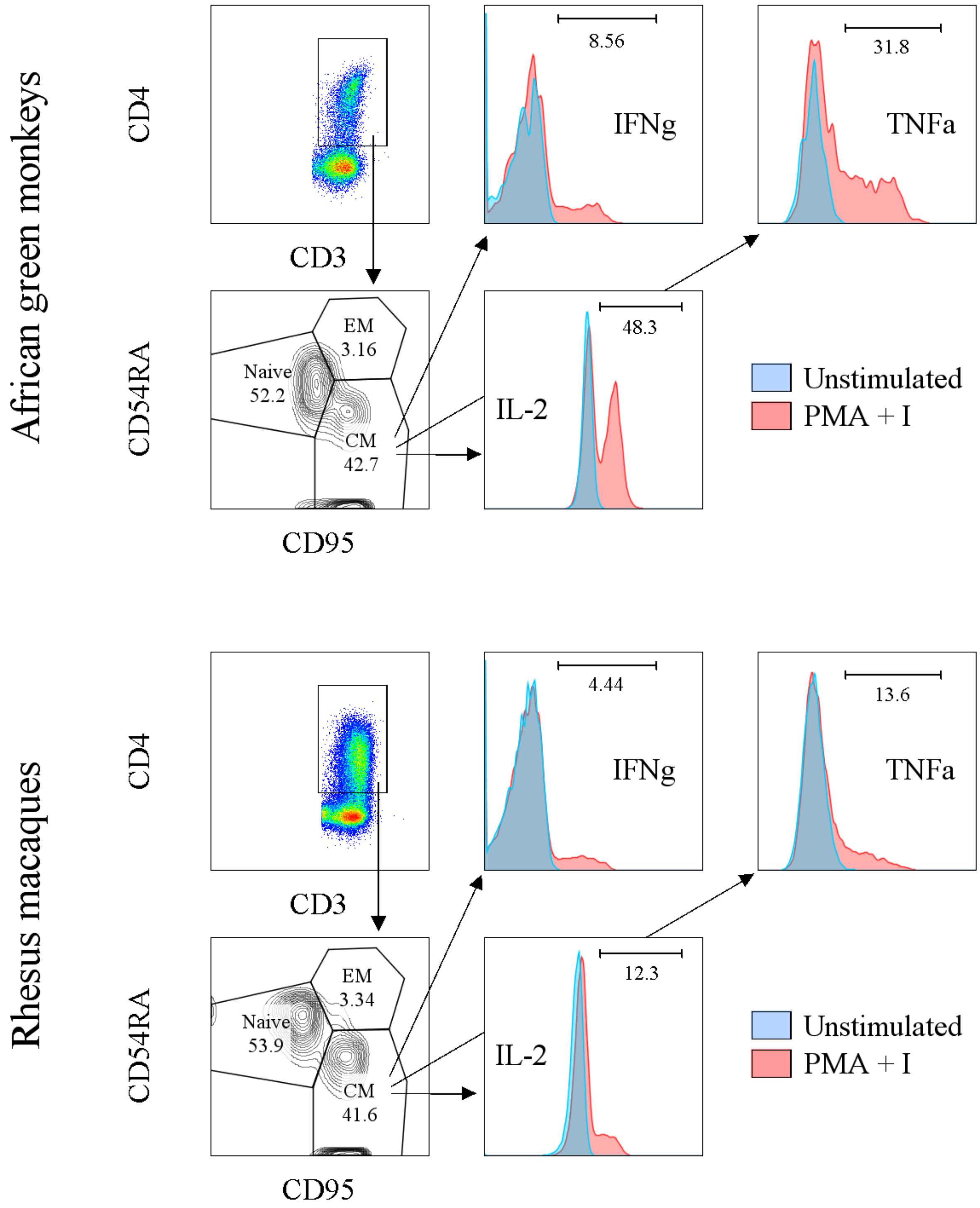

Figure S8. Cytokine-secretion patterns of central memory CD4^{+} T cells of African green monkeys (n = 8) and rhesus macaques (n = 19) after mitogenic stimulation. IFN: gamma interferon; IL2: interleukin 2; TNF: tumour necrosis factor alpha.

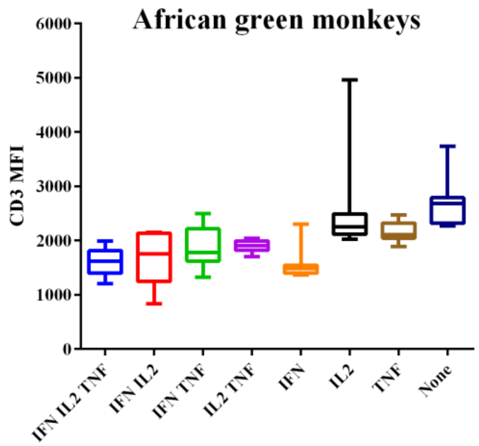

p -value	IFN IL2 TNF	IFN IL2	IFN TNF	IL2 TNF	IFN	IL2	TNF
IFN IL2 TNF							
IFN IL2	0.5920						
IFN TNF	0.1210	0.5077					
IL2 TNF	0.0273	0.2244	0.8404				
IFN	0.5683	0.5545	0.1404	0.0135			
IL2	0.0237	0.0267	0.0371	0.0920	0.0309		
TNF	0.0013	0.0341	0.0535	0.0116	0.0028	0.2701	
None	0.0004	0.0120	0.0004	0.0027	0.0014	0.5995	0.0165

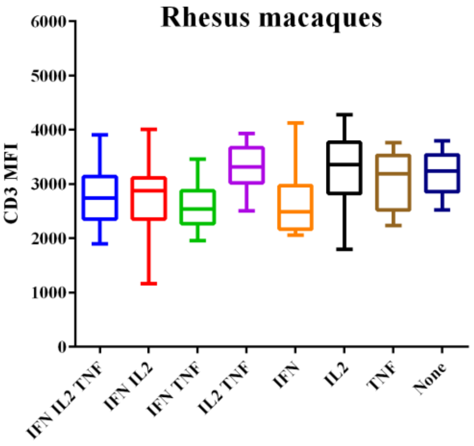

p -value	IFN IL2 TNF	IFN IL2	IFN TNF	IL2 TNF	IFN	IL2	TNF
IFN IL2 TNF							
IFN IL2	0.6111						
IFN TNF	0.0836	0.0944					
IL2 TNF	0.0000	0.0002	0.0000				
IFN	0.4295	0.2572	0.3707	0.0000			
IL2	0.0001	0.0000	0.0000	0.9749	0.0004		
TNF	0.0025	0.0323	0.0000	0.0059	0.0007	0.0329	
None	0.0000	0.0009	0.0000	0.0439	0.0000	0.1695	0.0547

Figure S9. Representative dot plots showing gating strategies used to delineate cytokine-secreting total T cells and CD4$^+$ T cells of cynomolgus macaques. Shown data are from an animal that has been infected with wild-type SIVmac239. The same gating method was applied to all cynomolgus macaques samples, including those infected with wild-type (n = 4) or Δnef (n = 4) viruses. PMA + I: 12-myristate 13-acetate and ionomycin; IFNg: gamma interferon; IL-2: interleukin 2; TNFa: tumour necrosis factor alpha.

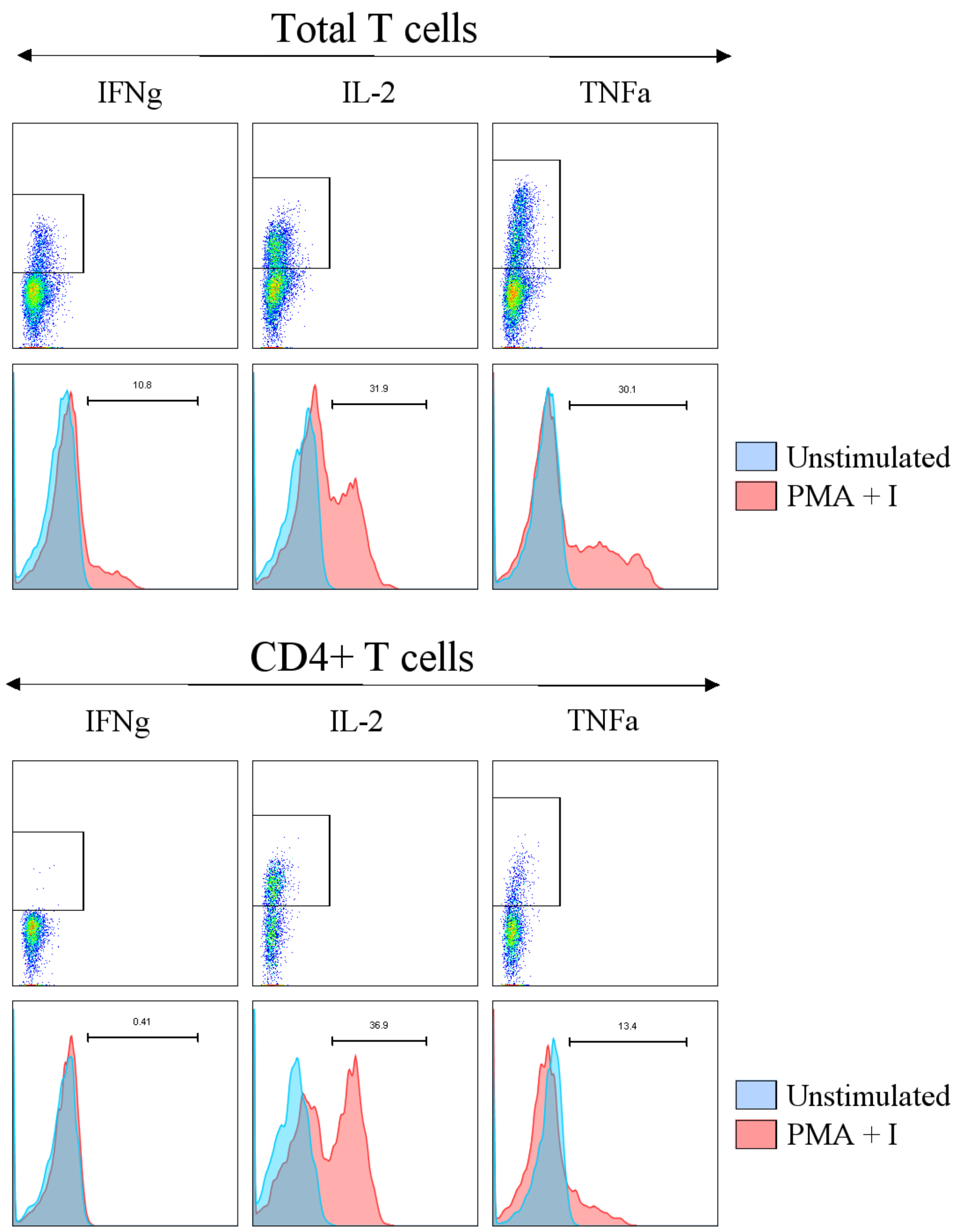

Supplement: Supplementary file 1 — Supplementary Information [file 41598_2019_41209_MOESM1_ESM.pdf]
